# Deep insights to explain the mechanism of carbon dot formation at various reaction times using the hydrothermal technique: FT-IR, ^13^C-NMR, ^1^H-NMR, and UV-visible spectroscopic approaches†

**DOI:** 10.1039/d3ra01646c

**Published:** 2023-05-10

**Authors:** Sewara J. Mohammed, Khalid M. Omer, Farouq E. Hawaiz

**Affiliations:** a Department of Chemistry, College of Science, University of Sulaimani Qlyasan Street Sulaimani 46002 Kurdistan Regional Government Iraq Sewara.mohammed@univsul.edu.iq; b Anesthesia Department, College of Health Sciences, Cihan University Sulaimaniya Sulaimaniya 46001 Kurdistan Region Iraq; c Department of Chemistry, College of Education, Salahaddin University – Hawler Erbil Kurdistan Iraq

## Abstract

A well-explained mechanism for synthesizing carbon dots (CDs) is not yet explored and is still a subject of great debate and challenge. This study used a one-step hydrothermal method to prepare highly efficient, gram-scale, excellent water solubility, and blue fluorescent nitrogen-doped carbon dots (NCDs) with the particle size average distribution of around 5 nm from 4-aminoantipyrine. The effects of varying synthesis reaction times on the structure and mechanism formation of NCDs were investigated using spectroscopic methods, namely FT-IR, ^13^C-NMR, ^1^H-NMR, and UV-visible spectroscopies. The spectroscopic results indicated that increasing the reaction time affects the structure of the NCDs. As the hydrothermal synthesis reaction time is extended, the intensity of the peaks in the aromatic region decreases, and new peaks in the aliphatic and carbonyl group regions are generated, which display enhanced intensity. In addition, the photoluminescent quantum yield increases as the reaction time increases. The presence of a benzene ring in 4-aminoantipyrine is thought to contribute to the observed structural changes in NCDs. This is due to the increased noncovalent π–π stacking interactions of the aromatic ring during the carbon dot core formation. Moreover, the hydrolysis of the pyrazole ring in 4-aminoantipyrine results in polar functional groups attached to aliphatic carbons. As the reaction time prolongs, these functional groups progressively cover a larger portion of the surface of the NCDs. After 21 h of the synthesis process, the XRD spectrum of the produced NCDs illustrates a broad peak at 21.1°, indicating an amorphous turbostratic carbon phase. The *d*-spacing measured from the HR-TEM image is about 0.26 nm, which agrees with the (100) plane lattice of graphite carbon and confirms the purity of the NCD product with a surface covered by polar functional groups. This investigation will lead to a greater understanding of the effect of hydrothermal reaction time on the mechanism and structure of carbon dot synthesis. Moreover, it offers a simple, low-cost, and gram-scale method for creating high-quality NCDs crucial for various applications.

## Abstract 2

The mechanism for synthesizing CDs remains unclear. This graphical abstract proposes a one-step hydrothermal method for CD formation from 4-aminoantipyrine, with varying reaction times. It illustrates the key steps involved in the synthesis process.

## Graphical abstract

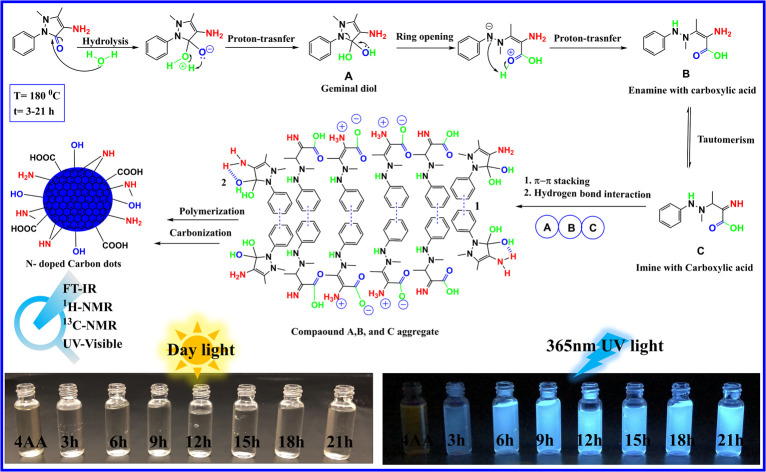

## 1. Introduction

Carbon dots (CDs) are carbon nanomaterials that have attracted considerable attention since their discovery in 2004 (ref. 1) owing to their low toxicity, excellent solubility, great biocompatibility, high chemical stability, numerous precursor sources, and distinctive photoluminescence (PL) properties.^2,3^ Carbon dots are often used in applications, including light emission,^4^ photocatalysis,^5^ biological imaging,^6^ and optoelectronic devices,^7^ such as LEDs^8^ and solar cells.^9^ Furthermore, CDs play an essential and exciting role in developing energy storage devices.^10^ Recent research has identified two types of CDs based on their solubility—aqueous soluble CDs^11^ and organic soluble CDs.^12^ Aqueous soluble CDs with good water solubility are widely studied in chemical and biological sensors, pharmaceutical and gene delivery, and bioimaging systems.^13–15^ CDs have been created using a variety of synthetic approaches from various sources of raw materials. The two primary methods for CD synthesis are top-down and bottom-up.^16^ In the top-down method, several techniques, such as electrochemical oxidation, laser ablation, chemical oxidation, arc discharge, and other physical methods, are usually used to generate CDs by breaking or peeling bulky carbon-rich structures.^17,18^ In the bottom-up method, a variety of natural^19^ and small molecules,^20^ such as citric acid,^21^l-ascorbic acid,^22^ ethylene diamine,^23^ amino acids,^24^ saccharides,^25,26^ glycerol,^27^ urea with malonic acid,^28^ thiourea,^29^ amino-aniline derivatives,^30^ citric acid with amino compounds,^31,32^ phloroglucinol and boric acid,^33^ and aldehydes,^34^ are used as precursors to produce CDs through combustion methods, microwave heating, hydrothermal synthesis, electrochemical synthesis, ultrasonic synthesis, and pyrolysis.^35^ The hydrothermal technique is now the most desirable because it easily uses large-scale synthesis. It does not need severe synthetic conditions, a one-step route, and low cost. Generally, the product of synthesis of CDs is often a complex mixture of compounds. Numerous techniques have been employed to separate and purify CDs, such as dialysis, centrifugation, solvent extraction, filtration, electrophoresis, chromatography, and combination methods.^36–38^ The precursors and reaction conditions affect the variations of surface groups. Broadly, the CDs contain a core comparable to graphite, either amorphous or crystalline, as has been suggested by numerous researchers.^39–42^ Others have proposed an amorphous core made entirely of sp^3^ carbon or one with an sp^3^–sp^2^ carbon ratio with a range of polar groups on its surface.^43–45^ The polar groups possessing carboxyl, hydrophilic hydroxyl, or amino groups on their surface are the most common. There is a significant difference in the fluorescence properties depending on the composition of CDs and their surface chemical groups. Many factors have been reported, such as the effect of precursors,^46^ type of solvent,^45^ reaction temperatures,^47^ and the ratio of the starting materials.^48^ There is very little research published evaluating the effects of reaction time to monitor the development of their properties.^49^ Despite the rapid growth in CDs research, there is a limited focus on understanding the synthesis mechanism, and the exact formation and structure of CDs remain unclear.^15,36,50,51^ According to the references and earlier publications, the postulated formation mechanism for CDs includes polymerization, nucleation, carbonization, and growth.^52^ Even though π–π stacking reactions are one of the most significant noncovalent interactions in aromatic systems, there has been no detailed study or debate to demonstrate the effect of this type of interaction on the mechanism of carbon dot formation.^53–56^ At present, some of the primary analytical tools, including electron microscopes and spectroscopy, are used to explore the structure and shape of CDs, but unfortunately, they could not cover the entire formation of these materials. Based on an intensive and extensive literature survey, few scientists employ FTIR and NMR spectrometers to examine the structure of CDs.^57,58^

Considering the above information, our goal is to investigate the macro preparation of novel NCDs with varying reaction times, their formation mechanism, and the factors that affect the increase in the quantum yield. In this work, based on the hydrolysis of cyclic amides and noncovalent π–π stacking interactions, highly water-soluble blue emission NCDs with high yield (40%) and quantum yield (18%) from 4-aminoantipyrine as a precursor by the hydrothermal method in deionized water at 180 °C with different the reaction times from 3–21 h have been synthesized. The mechanism and structure of NCDs formation have been investigated using spectroscopic techniques such as FT-IR, ^13^C-NMR, ^1^H-NMR, and UV-visible, which have not been widely utilized for this purpose previously.

## 2. Experimental work

### 2.1. Materials and chemicals

4-Aminoantipyrine was acquired from Sigma-Aldrich and used in its original form without any purification. All studies utilized deionized water, and chloroform was used to purify the NCDs.

### 2.2. Instrumental analyses and NCDs characterization

The FT-IR spectra were obtained in KBr pellets (*V*_max_ in cm^−1^) on a PerkinElmer spectrophotometer (Waltham, Massachusetts, USA). ^13^C-NMR and ^1^H-NMR spectra were measured in D_2_O on a Bruker DRX-500 MHz (Billerica, Massachusetts, USA). ^1^H-NMR chemical shifts were measured in parts per million (ppm) with respect to the residual solvent peak (D_2_O: ^1^H = 4.79 ppm). On a Cary 60 spectrophotometer (Agilent Technologies, USA) spectrometer, the UV-vis absorption spectra were collected. Photoluminescence (PL) emission measurements were performed using a Cary Eclipse fluorescence spectrophotometer (Agilent Technologies, USA). By comparing CDs to the reference fluorescein (*Φ* = 0.92), PLQY (*Φ*) was calculated. The X-ray diffraction (XRD) pattern of the CDs synthesized after 21 h was recorded on an Empyrean X-ray diffractometer (Panalytical, Netherlands). The morphology and microstructure of the CDs synthesized after 21 h was examined by high-resolution transmission electron microscopy (HRTEM) on an FEI TEC9G2, USA microscope with an accelerating voltage of 200 kV.

### 2.3. Hydrothermal synthesis of the NCDs

**Fig. 1:**
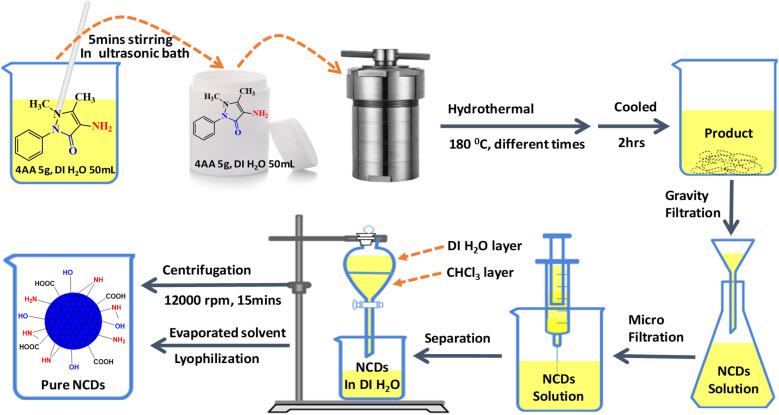
Schematic diagram of the synthesis and purification of NCDs from 4-aminoantipyrine.

The NCDs were synthesized from 4-aminoantipyrine (4AA) in deionized water by the hydrothermal method. In a 100 mL beaker, 4AA (5.0 g) was dissolved in 50 mL deionized water and then sonicated for 5 min until a clear pale yellow-colored solution was obtained. This solution was placed in a Teflon-lined autoclave (100 mL capacity) and heated at 180 °C in an oven with varying reaction times (3, 6, 9, 12, 15, 18, and 21 h). After naturally cooling to room temperature, the solution was filtered using a Whatman filter paper. A grade 93 with a pore size of (10 μm) was used to separate the dark-colored oil residue in the solutions. Then, the remaining large particles were removed through a disposable microporous membrane (0.22 μm) to obtain a yellow solution. After that, the extraction method was performed 3 times through a chloroform solvent to remove unreacted 4AA molecules. The aqueous layer was centrifuged (12 000 rpm, 30 min) to remove high-weight carbon aggregates. It evaporated the solvent using a rotatory evaporator and dried by lyophilization. Finally, 2 g of highly viscous honey-colored NCDs was obtained at 21 h for further characterization and stored at room temperature (Fig. 1).

## 3. Results and discussion

### 3.1. Synthesis and mechanism of the NCDs

**Fig. 2:**
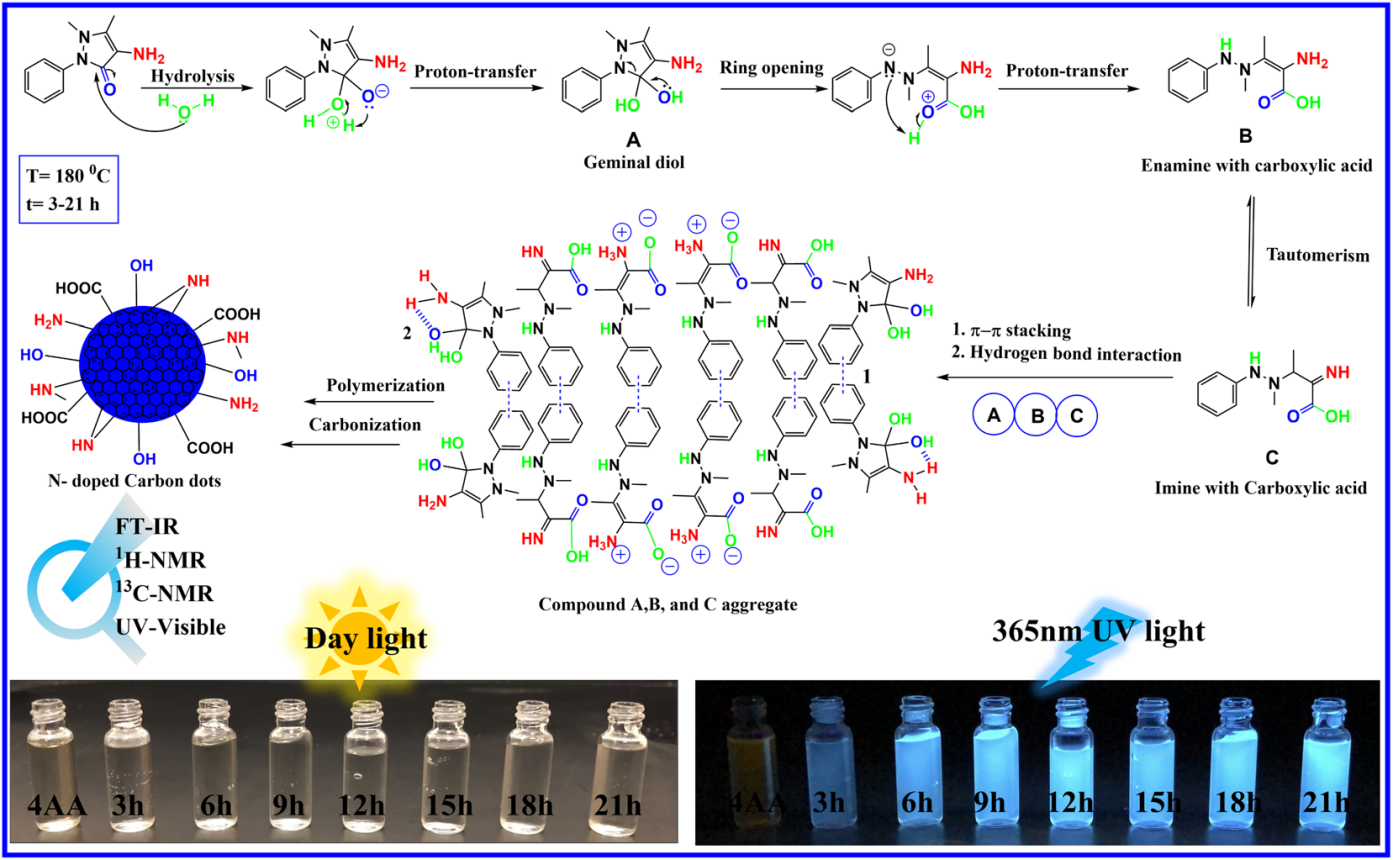
Proposed mechanism of NCDs formation.

The information gathered from spectroscopic results, combined with the high-water solubility of NCDs, attributed to several polar groups on the surface of NCDs, including hydroxyl, carboxyl, and amino groups, suggests the following mechanism, as depicted in Fig. 2. In the initial steps, the carbonyl group of 4-aminoantipyrine is attacked by the lone pair of oxygen atoms in the water molecule acting as a nucleophile, which is followed by the transfer of a hydrogen atom to give a geminal diol compound A. In the next step, due to the unstable geminal diol and the high temperatures present, the pyrazole ring in compound A opens and is followed by the proton transfer to produce an enamine with carboxylic acid compound B. On the one hand, compound B can undergo keto–enol tautomerism to form imine with carboxylic acid compound C. Finally, because of the presence of the benzene aromatic ring structure in compounds A, B, and C, the carbon core structure can be created *via* noncovalent π–π stacking interaction following a sequence of reactions and hydrogen bond interactions.^47–49^ At the same time, several hydroxyl, carboxyl, and amino groups in A, B, and C can still be present after the creation of carbon cores. This results in the growth of surface states on the surface of the carbon dots. On the other hand, the presence of water in the reaction makes the condensation reaction difficult because water is a protic solvent, which tends to donate protons to the reactants to reduce the water elimination reaction due to a decrease in the conjugation length. At the same time, the hydroxyl, carboxyl, and amino groups increase with reaction time.

### 3.2. FT-IR characterization of the NCDs

**Fig. 3:**
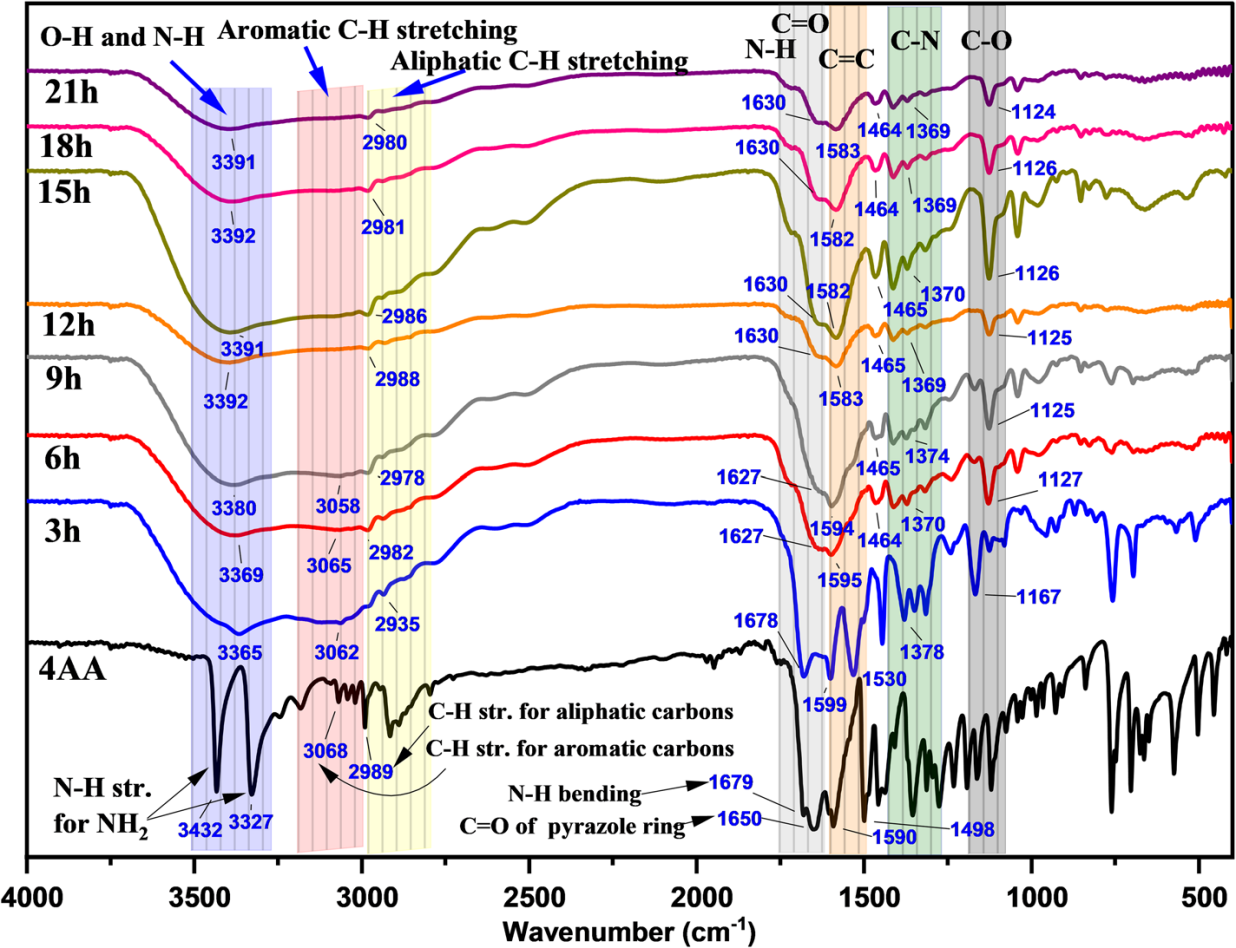
FT-IR spectra of 4AA and NCDs at varying synthesis reaction times.

In Fig. 3 and S1(a–h) (ESI†), the Fourier transform infrared (FT-IR) spectra of NCDs with synthesis times of 3, 6, 9, 15, 18, and 21 h are presented to investigate the reaction process and the presence of functional groups on the surface of NCDs. In the FT-IR spectrum of 4-aminoantipyrine (Fig. S1a† and 3), there are doublet peaks at 3432 and 3327 cm^−1^, a shoulder peak at 1679 cm^−1^, and a medium peak at 1655 cm^−1^, which can be assigned to the NH_2_ stretching of 4AA, N–H bending of 4AA, and carbonyl group (C=O) group stretching of the pyrazole ring, respectively. Along with the reaction, these peaks disappear, and instead, other new peaks of N–H stretching of amine or/and O–H stretching alcohol at 3391 cm^−1^,^41,42^ broadband of O–H stretching of carboxylic acid^59^ at about 3500–2500 cm^−1^, a shoulder peak of N–H bending of amine at 1630 cm^−1^,^60,61^ and C=O of carboxylic acid stretching at 1590 cm^−1^ appeared.^62^ The changes observed suggest that the pyrazole ring undergoes the process of hydrolysis to form NCDs that have both a carboxyl and an amine group. Moreover, the FTIR spectra of NCDs are very similar to those of amino acids during comparison, particularly serine and d-threonine (Fig. S2(a and b); ESI†), which strongly suggests the presence of both carboxylic and amine groups on their surface. This can be attributed to the fact that the C=O in amino acids has a lower frequency compared to C=O in carboxylic acids that do not contain amines due to resonance (more single-bond character) and the intramolecular hydrogenation between amino and carboxylic acid groups.^63,64^ In addition, two new peaks at 1370 and 1125 cm^−1^ gradually appear with increasing reaction time. These new peaks contribute to C–N and C–O group formation in the NCDs.^65^

Furthermore, the intensity peaks of C–H stretching of the aromatic ring in the range of 3068 to 3018 cm^−1^ gradually decrease with increasing reaction time. The change proposes the influence of the π–π stacking interaction of the aromatic ring on the creation of carbon cores in NCDs. Overall, the evaluation of the above FT-IR spectra illustrated how some modifications occurred during the synthesis process, revealing the formation of highly polar functional groups, including hydroxyl, amine, and carboxyl groups, on the surface of the NCDs. These findings are consistent with other observations, such as the solubility in water and the luminous properties.

### 3.3. ^13^C-NMR and ^1^H-NMR characterization of the NCDs

**Fig. 4:**
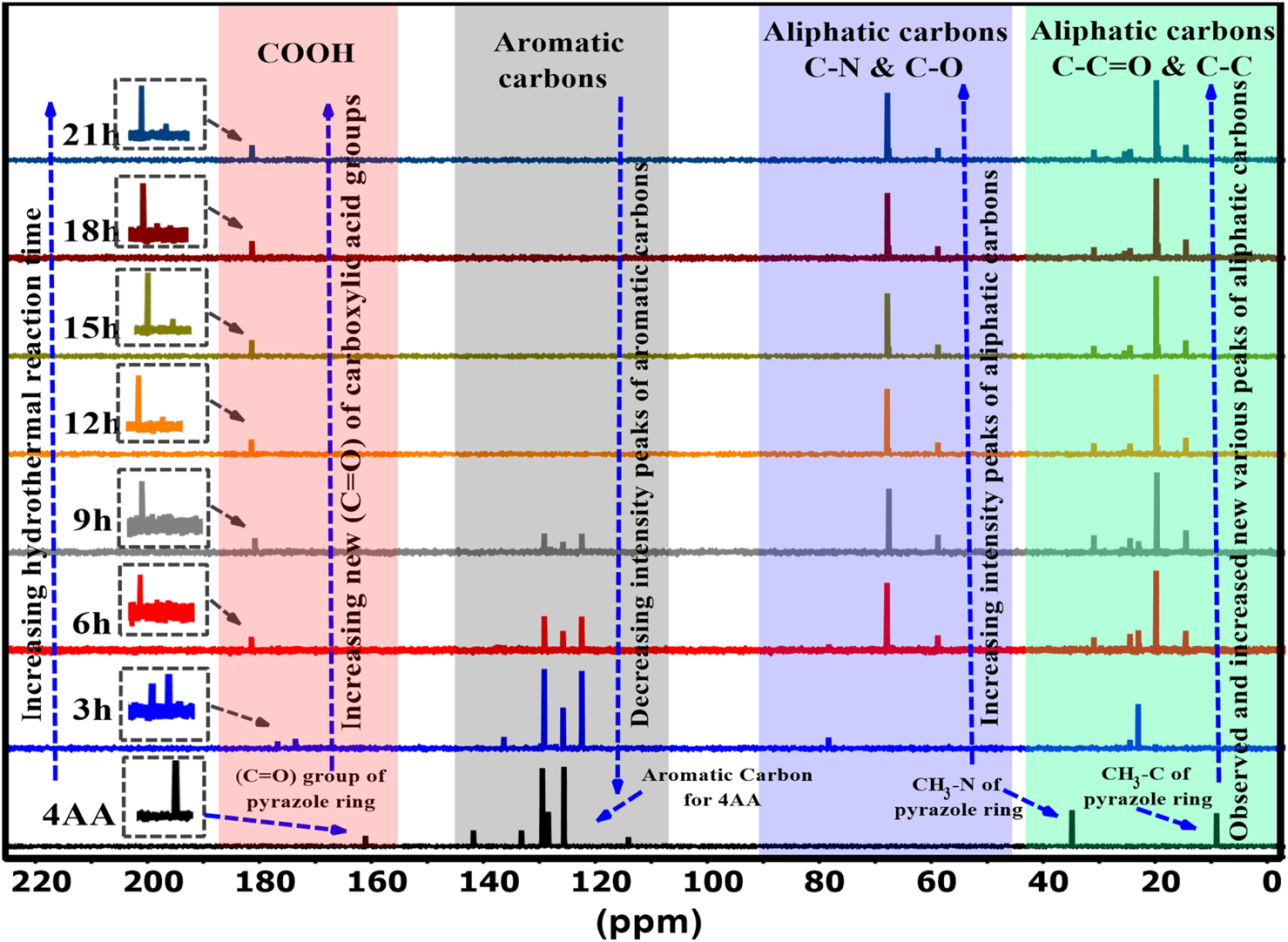
^13^C-NMR spectra of 4AA and NCDs at different synthesis reaction times.

Nuclear magnetic resonance spectroscopy (NMR) characterization of 4AA and NCDs with synthesis times of 3, 6, 9, 15, 18, and 21 h were performed in a deuterium oxide (D_2_O) solvent. The ^13^C-NMR spectra of NCDs are displayed in Fig. 4 and S3(a–h) (ESI†). In the ^13^C-NMR spectra of 4-aminoantipyrine (Fig. 4 and S3a†), a peak at 161 ppm is attributed to the carbonyl (C=O) group of the pyrazole ring. As the reaction time increased, this peak disappeared, suggesting that the basic structure of 4AA was not retained, and multiple resonance peaks appeared in the range of 174–181 ppm, demonstrating the formation of various carboxyl functional groups. At reaction times of 12, 15, 18, and 21 h, more carboxyl resonance peaks appeared, revealing that this stage is the intermediate period of carbon dot generation and the reaction gradually reached completion and eventually produced stable carboxylic acid functional groups. In addition, as the reaction proceeded, the peaks of the carbon aromatic ring at 141–114 ppm gradually weakened and eventually disappeared with the combined FT-IR spectra; it is speculated to be caused by an increase in the π–π stacking interaction^56^ of the aromatic ring during the formation of the carbon dot core,^24^ with the surface of the NCDs covered by polar functional groups attached to the aliphatic carbons. Moreover, the new collection peaks with various intensities were observed from 67–58 ppm, corresponding to the aliphatic carbons attached with amine, hydroxyl, and carboxyl groups in NCDs formed during the carbonization process. The increase in these peaks supports the hypothesis, and the findings agree with the FT-IR results. Finally, due to the effect of carboxyl, beta-amino, and beta-hydroxyl groups, other collection peaks with various intensities were observed in the range of 30–14 ppm assigned to the aliphatic carbon groups, but 4AA gives two peaks at 34 and 9 ppm, indicating that the basic structure of 4AA is not retained. The analysis of the ^13^C-NMR data indicated that some changes took place during the synthesis process, demonstrating the creation of highly polar functional groups such as hydroxyl, amine, and carboxyl groups on the surface of the NCDs. The ^13^C-NMR spectra also revealed that as the reaction progresses, the intensity peaks of the carbon in the aromatic region decrease, and new carbon peaks in the aliphatic and carbonyl group regions are formed, which display enhanced intensity. The findings of the water solubility and luminescence properties were consistent with the results of the ^13^C-NMR analysis. The results demonstrate that utilizing both ^13^C-NMR and FTIR spectroscopy gives a more in-depth and precise insight into the formation and structure of carbon dots.

**Fig. 5:**
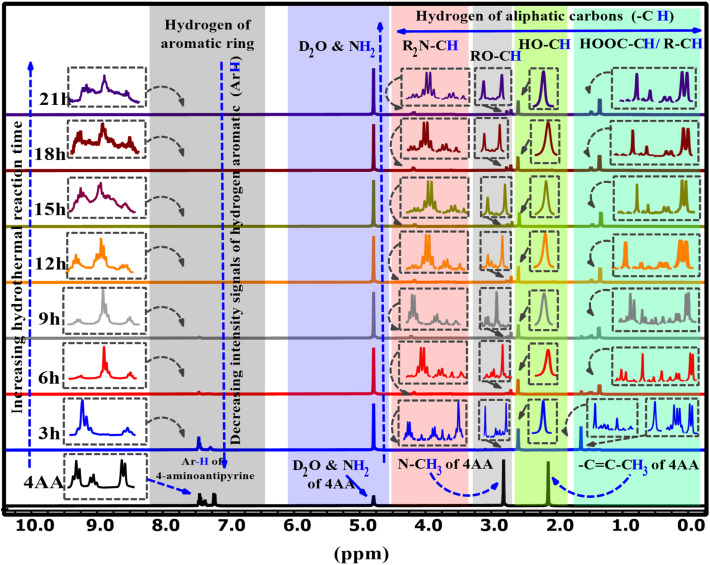
^1^H-NMR spectra of 4AA and NCDs at different synthesis reaction times.

**Fig. 6:**
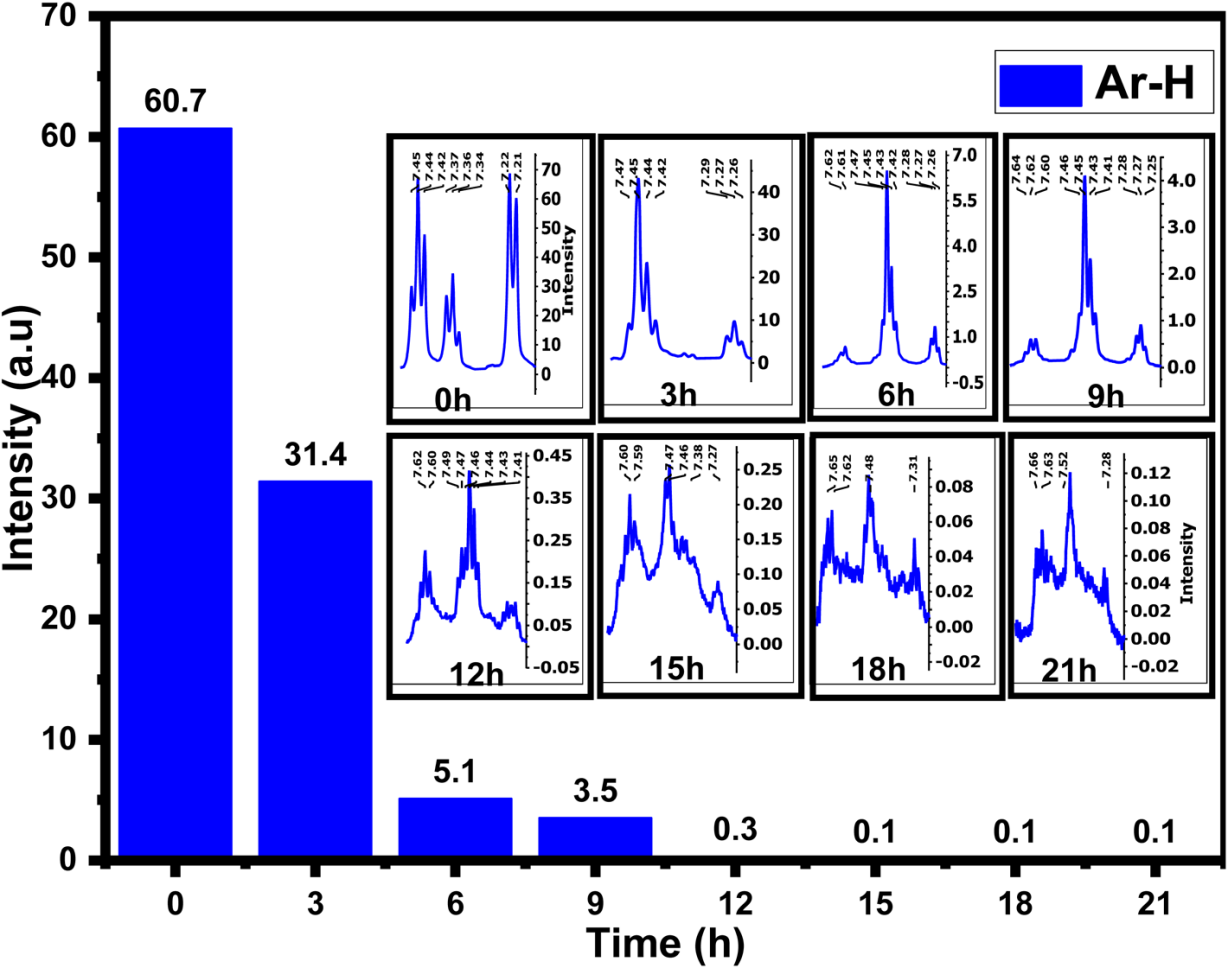
Comparison of hydrogen aromatic ring intensity signals for 4AA and NCDs at different times.

The ^1^H-NMR spectra of 4AA and NCDs with synthesis times of 3, 6, 9, 15, 18, and 21 h are shown in Fig. 5 and S4(a–h) (ESI†). ^1^H-NMR findings, concurring with ^13^C-NMR and FT-IR results, suggest combining ^1^H-NMR with ^13^C-NMR and FT-IR spectroscopy in a clearer and more in-depth examination of the structure and mechanism of carbon dot formation. Initially, for 4-aminoantipyrine and each of the different synthesis reaction times, the hydrogen of both carboxylic acid (COOH) and amino (NH_2_) groups scrambled with D_2_O, and their signals are not observed, but their effects have been seen in other ranges.^66^ In addition, one of the most significant signals is for the hydrogen of aromatic rings in the range of 8–7 ppm, which is precisely supported by FT-IR or ^13^C-NMR spectra. The ^1^H-NMR spectrum of 4-aminoantipyrine (Fig. 5 and S4a†) showed that the intensity peaks of the hydrogen aromatic ring at 7.45–7.21 ppm gradually decreased and eventually nearly disappeared as the reaction time increased, suggesting that the benzene ring in 4-aminoantipyrine was carbonized and formed NCDs under high temperature and high pressure. Fig. 6 clearly illustrates, along with the reaction time, that the shape of the signals of the hydrogen aromatic ring changes and the intensity signals of the hydrogen aromatic ring decreased; initially, the intensity signals of the hydrogen aromatic ring was 60.7, which decreased to 31.4, 5.1, and 3.5 after 3, 6, and 9 h, respectively.

Moreover, at 12, 15, 18, and 21 h, the signals of the hydrogen aromatic ring approach zero and become rather invisible. In addition, as the reaction time increased, the collection hydrogen signals with various intensities were obtained in the range of 4.24–1.4 ppm, which were new signals that corresponded to the various hydrogen of aliphatic carbons attached with amine, ether, hydroxyl, carboxylic acid, and aliphatic groups, respectively,^36^ on the surface of NCDs. The peaks in the NCDs spectra are different from those in the 4-amnioantipyrine spectrum, suggesting that the carbon dots have a distinct structure. The increase in these peaks confirmed this hypothesis and the types of functional groups present on the surface of NCDs. The results agree with the FT-IR and ^13^C-NMR characterization analyses.

### 3.4. Optical properties of NCDs

**Fig. 7:**
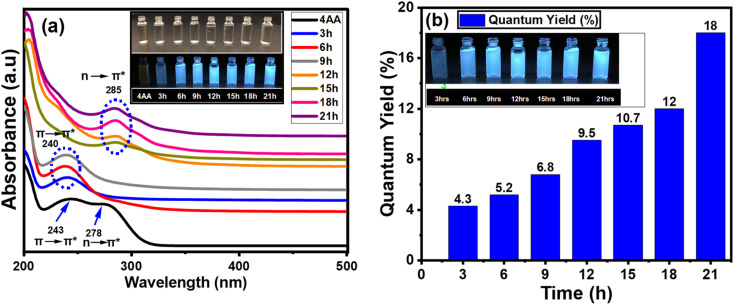
(a) Absorption spectra 4AA and NCDs at different times; inset: photographs of NCDs taken in daylight (up) and an ultraviolet beam of 365 nm (down). (b) Photoluminescent quantum yield values of NCDs.

In addition to the FT-IR and NMR spectra, the light absorption and emission sources were examined. The variation in the UV-vis absorption spectra could be utilized to detect changes in the surface functionality. The results obtained from UV-visible spectroscopy agree with those from FT-IR, ^13^C-NMR, and ^1^H-NMR analyses. This indicates that a more comprehensive and precise understanding of the structure and process of carbon dot formation can be achieved using a combination of UV-visible, FT-IR, ^13^C-NMR, and ^1^H-NMR spectroscopy. Fig. 7(a, b) and S5(a–g) (ESI†) show the absorption, PLE, and PL spectra of NCDs with synthesis times of 3, 6, 9, 15, 18, and 21 h. The effect of reaction time on UV-vis spectra of NCDs is shown in Fig. S5(a–g) (ESI†). The UV-visible spectrum of 4-aminoantipyrine (Fig. 7a) displayed two peaks—one at 243 nm, which corresponded to the π → π* transition of the (sp^2^ aromatic/alkenyl C=C bonds)^67^ and another absorption band at 278 nm, which is assigned to the n → π* transition of the C=O bond. After 3, 6, and 9 h of synthesis times, one peak is nearly identical, but there are some differences in the intensity at 240 nm corresponding to the π → π* transition of sp^2^ aromatic/alkenyl C=C bonds or C=N bonds in Fig. S5a–c,† and 7a.^68,69^ On the other hand, after 12, 15, 18, and 21 h, another peak was observed with some changes in the intensity at 285 nm, which is attributed to n → π* transition by nonbonding orbitals such as C=O bonds^70^ (Fig. S5d–f,† and 7a). This is in good agreement with the results of the chemical composition analysis, especially the FT-IR, ^13^C-NMR, and ^1^H-NMR data, which shows that the proportion of the carboxyl group increases with reaction time.

Although absorption measurements offer insights into transitions between the ground and excited states, photoluminescence excitation (PLE) reveals information about the energy levels associated with the specific light emission bands. After 3 h of the synthesis process (Fig. S5a†), the PLE spectra showed one PLE band at 320 nm, while for all the other times, another PLE band at 340 nm can be seen in Fig. S5(b–g).† These differences are caused by the presence of nearly identical types of emission sites on their surface. Furthermore, the PL emission at each time is placed in the blue region of the electromagnetic spectrum at ∼425 nm (Fig. S5(a–g)†).

The PL emission spectra of NCDs with synthesis times were obtained using excitation wavelengths changing from 340 to 400 nm with a step of 10 nm (Fig. S6, ESI†). After 3 h, the PL emission spectra of the synthesis process (Fig. S6a†) displayed the peak position excitation-independent emission. For all other synthesis times, the PL emission spectra are nearly the same and confirm that the NCDs clearly show excitation-independent emission^71–73^ except for excitation with 340 and 350 nm, where the PL peak positions are excitation-dependent. This characteristic is similar to inorganic quantum dots and will be advantageous for creating a pure-color light-emitting device.

The photoluminescent quantum yield (PLQY) is an accurate indicator of fluorescent materials' efficiency. The comparison approach of Williams *et al.* is the most reliable formula for calculating the PLQY. Fig. 7b shows the PLQY of NCDs, where values increase proportionally with reaction time.

### 3.5. HR-TEM, XRD, and SAED of NCDs

**Fig. 8:**
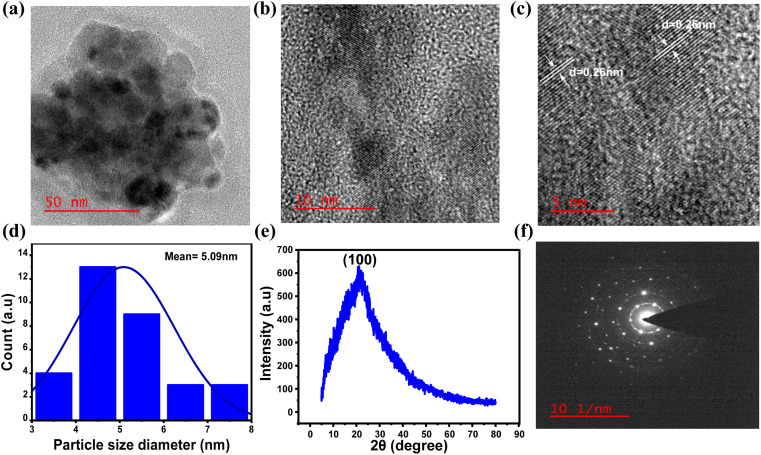
HRTEM images with different magnifications of CDs in (a) 50 nm, (b) 10 nm, and (c) 5 nm showing lattice fringes, (d) particle size distribution histogram as calculated from (a) and *n* = 30, (e) XRD pattern and (f) SAED pattern of NCDs with synthesis time of 21 h.

To gain a deeper understanding of the formation of N-doped carbon dots from 4AA, further investigations were carried out, with a particular emphasis on the 21 h duration during the synthesis process, when the quantum yield value was observed to be at its highest. The investigations involved high-resolution transmission electron microscopy (HR-TEM), X-ray diffraction (XRD), and selected area electron diffraction (SAED), as shown in Fig. 8a–f. The results obtained from these characterizations are consistent with the findings from spectroscopic and chemical composition investigations. HR-TEM exhibited that NCDs had an average particle size distribution of about 5 nm (Fig. 8d). In addition, the lattice fringes with a *d*-spacing measured from the HRTEM image (Fig. 8c) is about 0.26 nm, which agrees with the (100) plane lattice of graphitic carbon.^59–61^ On the other hand, the absence of 0.33 nm graphitic (002) spacing suggests that the surface of NCDs is covered by polar functional groups attached to aliphatic carbons.^74,75^ In addition, the crystal structure of the NCDs has been examined through X-ray diffraction (XRD) analysis. The XRD pattern (Fig. 8e) exhibited a broad peak at 21.1°, ascribed to the turbostratic carbon phase.^76^ The NCDs exhibit an interlayer spacing (*d*) of 0.42 nm, indicating a more open and irregular structure than graphite and nanodiamonds with smaller interlayer spacing.^77^ In contrast, the structure resembles turbostratic graphite carbon, suggesting that the NCDs possess a low degree of crystallization, which is attributed to the higher occurrence of oxygen and nitrogen groups within the NCDs structure.^78,79^ Furthermore, the selected area electron diffraction (SAED) pattern of NCDs (Fig. 8f) shows amorphous nature, which is consistent with the XRD pattern (Fig. 8e).^50^

## 4. Conclusion

In conclusion, a new type of water-soluble, gram-scale, blue-fluorescent NCDs was synthesized *via* the hydrothermal method using 4-aminoantipyrine as a precursor, with an average particle size distribution of about 5 nm. The investigation of the effects of varying synthesis reaction times on the structure and mechanism formation of NCDs using spectroscopic techniques revealed that increasing the reaction time affects the structure of the NCDs by generating new peaks in the aliphatic and carbonyl group regions, increasing noncovalent π–π stacking interactions of the aromatic ring and covering a larger portion of the NCDs surface with polar functional groups attached to aliphatic carbons. Furthermore, the mechanism of forming NCDs through the hydrothermal method was proposed based on the spectroscopic results and literature. Initially, 4-aminoantipyrine goes through several reactions such as hydrolysis, ring opening, and keto–enol tautomerism. As a result, various compounds with different types of polar functional groups are produced. After that, these compounds interact through noncovalent π–π stacking and hydrogen bond interactions, leading to polymerization and forming a carbon core. Finally, NCDs with abundant surface polar functional groups were gradually produced as the reaction time increased. This study contributes to a better understanding of the mechanism of carbon dot formation and offers a simple, low-cost, and gram-scale method for creating high-quality NCDs, which is crucial for various applications.

## Author contributions

Sewara J. Mohammed performed the artificial experiments, analyzed the data, and wrote the manuscript while directing the project. Farouq E. Hawaiz provided consultation, reviewed the work, and offered direction throughout the project. Khalid M. Omar contributed to the project by reviewing the manuscript.

## Conflicts of interest

The authors declare no conflict of interest.

## Supplementary Material

RA-013-D3RA01646C-s001

## References

[cit1] Xu X., Ray R., Gu Y., Ploehn H. J., Gearheart L., Raker K., Scrivens W. A. (2004). J. Am. Chem. Soc..

[cit2] Georgakilas V., Perman J. A., Tucek J., Zboril R. (2015). Chem. Rev..

[cit3] Liu M. L., Bin Chen B., Li C. M., Huang C. Z. (2019). Green Chem..

[cit4] Yan F., Sun Z., Zhang H., Sun X., Jiang Y., Bai Z. (2019). Microchim. Acta.

[cit5] Han M., Zhu S., Lu S., Song Y., Feng T., Tao S., Liu J., Yang B. (2018). Nano Today.

[cit6] Hong G., Diao S., Antaris A. L., Dai H. (2015). Chem. Rev..

[cit7] Feng T., Tao S., Yue D., Zeng Q., Chen W., Yang B. (2020). Small.

[cit8] Zhang Y., Xiao J., Zhuo P., Yin H., Fan Y., Liu X., Chen Z. (2019). ACS Appl. Mater. Interfaces.

[cit9] Gao N., Huang L., Li T., Song J., Hu H., Liu Y., Ramakrishna S. (2020). J. Appl. Polym. Sci..

[cit10] Zhai Y., Zhang B., Shi R., Zhang S., Liu Y., Wang B., Zhang K., Waterhouse G. I. N., Zhang T., Lu S. (2022). Adv. Energy Mater..

[cit11] Lu W., Gong X., Yang Z., Zhang Y., Hu Q., Shuang S., Dong C., Choi M. M. F. (2015). RSC Adv..

[cit12] Wu M., Zhan J., Geng B., He P., Wu K., Wang L., Xu G., Li Z., Yin L., Pan D. (2017). Nanoscale.

[cit13] Tejwan N., Kundu M., Ghosh N., Chatterjee S., Sharma A., Abhishek Singh T., Das J., Sil P. C. (2022). Inorg. Chem. Commun..

[cit14] Li L., Lu C., Li S., Liu S., Wang L., Cai W., Xu W., Yang X., Liu Y., Zhang R. (2017). J. Mater. Chem. B.

[cit15] Liu J., Li R., Yang B. (2020). ACS Cent. Sci..

[cit16] Xia C., Zhu S., Feng T., Yang M., Yang B. (2019). Adv. Sci..

[cit17] Wang Y., Hu A. (2014). J. Mater. Chem. C.

[cit18] Xiao L., Sun H. (2018). Nanoscale Horiz..

[cit19] Ali H. R. H., Hassan A. I., Hassan Y. F., El-Wekil M. M. (2020). Anal. Bioanal. Chem..

[cit20] Sharma A., Das J. (2019). J. Nanobiotechnol..

[cit21] Liu C., Cheng R., Guo J., Li G., Li H., Ye H. G., Bin Liang Z., Wang C. F., Chen S. (2021). Chin. Chem. Lett..

[cit22] Cailotto S., Amadio E., Facchin M., Selva M., Pontoglio E., Rizzolio F., Riello P., Toffoli G., Benedetti A., Perosa A. (2018). ACS Med. Chem. Lett..

[cit23] Zhang T., Gong X., Zhang Y. (2023). Arabian J. Chem..

[cit24] Pandit S., Behera P., Sahoo J., De M. (2019). ACS Appl. Bio Mater..

[cit25] Omer K. M., Hama Aziz K. H., Mohammed S. J. (2019). New J. Chem..

[cit26] Aziz S. B., Hassan A. Q., Mohammed S. J., Karim W. O., Kadir M. F. Z., Tajuddin H. A., Chan N. N. M. Y. (2019). Nanomaterials.

[cit27] Lai C. W., Hsiao Y. H., Peng Y. K., Chou P. T. (2012). J. Mater. Chem..

[cit28] Fan Y. Z., Zhang Y., Li N., Liu S. G., Liu T., Li N. B., Luo H. Q. (2017). Sens. Actuators, B.

[cit29] Kumari R., Sahu S. K. (2020). Langmuir.

[cit30] Liu C., Ning D., Zhang C., Liu Z., Zhang R., Zhao J., Zhao T., Liu B., Zhang Z. (2017). ACS Appl. Mater. Interfaces.

[cit31] Schneider J., Reckmeier C. J., Xiong Y., Von Seckendorff M., Susha A. S., Kasak P., Rogach A. L. (2017). J. Phys. Chem. C.

[cit32] Deng M., Wang S., Liang C., Shang H., Jiang S. (2016). RSC Adv..

[cit33] Niu X., Song T., Xiong H. (2021). Chin. Chem. Lett..

[cit34] Bhattacharya S., Phatake R. S., Nabha Barnea S., Zerby N., Zhu J. J., Shikler R., Lemcoff N. G., Jelinek R. (2019). ACS Nano.

[cit35] Li L., Dong T. (2018). J. Mater. Chem. C.

[cit36] Gude V., Das A., Chatterjee T., Mandal P. K. (2016). Phys. Chem. Chem. Phys..

[cit37] Bhattacharya D., Mishra M. K., De G. (2017). J. Phys. Chem. C.

[cit38] Hinterberger V., Damm C., Haines P., Guldi D. M., Peukert W. (2019). Nanoscale.

[cit39] Kelarakis A. (2014). MRS Energy Sustain..

[cit40] Martindale B. C. M., Hutton G. A. M., Caputo C. A., Prantl S., Godin R., Durrant J. R., Reisner E. (2017). Angew. Chem., Int. Ed..

[cit41] Ansi V. A., Renuka N. K. (2019). J. Lumin..

[cit42] Wei S., Yin X., Li H., Du X., Zhang L., Yang Q., Yang R. (2020). Chem.–Eur. J..

[cit43] Xu Q., Cai W., Zhang M., Su R., Ye Y., Li Y., Zhang L., Guo Y., Yu Z., Li S., Lin X., Chen Y., Luo Y., Street J., Xu M. (2018). RSC Adv..

[cit44] Tepliakov N. V., Kundelev E. V., Khavlyuk P. D., Xiong Y., Leonov M. Y., Zhu W., Baranov A. V., Fedorov A. V., Rogach A. L., Rukhlenko I. D. (2019). ACS Nano.

[cit45] Mintz K. J., Bartoli M., Rovere M., Zhou Y., Hettiarachchi S. D., Paudyal S., Chen J., Domena J. B., Liyanage P. Y., Sampson R., Khadka D., Pandey R. R., Huang S., Chusuei C. C., Tagliaferro A., Leblanc R. M. (2021). Carbon.

[cit46] Hasan M. R., Saha N., Quaid T., Toufiq Reza M. (2021). Energies.

[cit47] Ogi T., Aishima K., Permatasari F. A., Iskandar F., Tanabe E., Okuyama K. (2016). New J. Chem..

[cit48] Zhang Y., Liu X., Fan Y., Guo X., Zhou L., Lv Y., Lin J. (2016). Nanoscale.

[cit49] Papaioannou N., Titirici M. M., Sapelkin A. (2019). ACS Omega.

[cit50] De B., Karak N. (2013). RSC Adv..

[cit51] Mucha S. G., Firlej L., Bantignies J. L., Żak A., Samoć M., Matczyszyn K. (2020). RSC Adv..

[cit52] Han B., Hu X., Zhang X., Huang X., An M., Chen X., Zhao D., Li J. (2022). RSC Adv..

[cit53] Pérez E. M., Martín N. (2015). Chem. Soc. Rev..

[cit54] Yang D., Gao S., Fang Y., Lin X., Jin X., Wang X., Ke L., Shi K. (2018). Nanomedicine.

[cit55] Cockroft S. L., Hunter C. A., Lawson K. R., Perkins J., Urch C. J. (2005). J. Am. Chem. Soc..

[cit56] Duan P., Li X., Wang T., Chen B., Juhl S. J., Koeplinger D., Crespi V. H., Badding J. V., Schmidt-Rohr K. (2018). J. Am. Chem. Soc..

[cit57] Tang H., Tang Y., Xiao M., Zhu H., Guo M. (2022). Colloids Surf., A.

[cit58] Arroyave J. M., Ambrusi R. E., Robein Y., Pronsato M. E., Brizuela G., Di Nezio M. S., Centurión M. E. (2021). Appl. Surf. Sci..

[cit59] Zhang L., Luo X., Qin Y., Li Y. (2017). RSC Adv..

[cit60] Yan Y., Liu J. H., Li R. S., Li Y. F., Huang C. Z., Zhen S. J. (2019). Anal. Chim. Acta.

[cit61] Lampman D. L. P. G. M. G. S. K. and Vyvyan J. R., Introduction to Spectroscopy, 5th edn, 2013

[cit62] Li P., Sun X. Y., Shen J. S., Liu B. (2016). RSC Adv..

[cit63] Gaddam R. R., Vasudevan D., Narayan R., Raju K. V. S. N. (2014). RSC Adv..

[cit64] Patir K., Gogoi S. K. (2019). Nanoscale Adv..

[cit65] Pawar S., Togiti U. K., Bhattacharya A., Nag A. (2019). ACS Omega.

[cit66] Zivkovic A., Bandolik J. J., Skerhut A. J., Coesfeld C., Zivkovic N., Raos M., Stark H. (2016). J. Chem. Educ..

[cit67] Liu H., Wang Q., Shen G., Zhang C., Li C., Ji W., Wang C., Cui D. (2014). Nanoscale Res. Lett..

[cit68] Li W., Zhang X., Miao C., Li R., Ji Y. (2020). Anal. Bioanal. Chem..

[cit69] Wu P., Li W., Wu Q., Liu Y., Liu S. (2017). RSC Adv..

[cit70] Manioudakis J., Victoria F., Thompson C. A., Brown L., Movsum M., Lucifero R., Naccache R. (2019). J. Mater. Chem. C.

[cit71] Zhu Q., Mao H., Li J., Hua J., Wang J., Yang R., Li Z. (2021). Spectrochim. Acta, Part A.

[cit72] Qu D., Zheng M., Li J., Xie Z., Sun Z. (2015). Light: Sci. Appl..

[cit73] Qu D., Sun Z., Zheng M., Li J., Zhang Y., Zhang G., Zhao H., Liu X., Xie Z. (2015). Adv. Opt. Mater..

[cit74] Hill S. A., Benito-Alifonso D., Morgan D. J., Davis S. A., Berry M., Galan M. C. (2016). Nanoscale.

[cit75] Tang L., Ji R., Li X., Bai G., Liu C. P., Hao J., Lin J., Jiang H., Teng K. S., Yang Z., Lau S. P. (2014). ACS Nano.

[cit76] Souza S. K. B. A. D., Suhail B. (2019). Int. Nano Lett..

[cit77] Pal A., Sk M. P., Chattopadhyay A. (2020). Mater. Adv..

[cit78] Hou J., Yan J., Zhao Q., Li Y., Ding H., Ding L. (2013). Nanoscale.

[cit79] Johra F. T., Lee J. W., Jung W. G. (2014). J. Ind. Eng. Chem..

