# Supplementary material for: Deep insights to explain the mechanism of carbon dot formation at various reaction times using the hydrothermal technique: FT-IR, 13C-NMR, 1H-NMR, and UV-visible spectroscopic approaches
Source: RSC Adv. 2023 May 10;13(21):14340–9. doi: 10.1039/d3ra01646c (PMC10170355; doi:10.1039/d3ra01646c)
Supplement: RA-013-D3RA01646C-s001 [file RA-013-D3RA01646C-s001.pdf]

Electronic Supplementary Material (ESI) for RSC Advances.
This journal is © The Royal Society of Chemistry 2023

Electronic Supplementary Information

Deep insights to explain the mechanism of carbon dot formation at various reaction times using the hydrothermal technique: FT-IR, ^{13}C-NMR, ^{1}H-NMR, and UV-Visible spectroscopic approaches

Sewara J. Mohammed,*[a,b] Khalid M. Omer, [b] and Farouq E. Hawaiz [c]

a. Department of Chemistry, College of Science, University of Sulaimani, Qlyasan Street, Sulaimani,46002, Kurdistan Regional Government, Iraq.

b. Anesthesia department, College of health sciences, Cihan University Sulaimaniya, Sulaimaniya 46001, Kurdistan Region, Iraq.

c. Department of Chemistry, College of Education, Salahaddin University – Hawler, Erbil-Kurdistan, Iraq

*Corresponding author. E-mail: Sewara.mohammed@univsul.edu.iq

1. **Fig. S1** FT-IR spectra of (a) 4AA, (b) 3h, (c) 6 h, (d) 9h, (e) 12h, (f) 15h, (g) 18h, and (h) 21h NCDs.

2. **Fig. S2** FT-IR spectra of (a) serine, and (b) d-threonine. We have found both FT-IR spectra from this site. (www.chemicalbook.com)

3. **Fig. S3** ^{13}C-NMR spectra of (a) 4AA, (b) 3h, (c) 6 h, (d) 9h, (e) 12h, (f) 15h, (g) 18h, and (h) 21h NCDs.

4. **Fig. S4** ^{1}H-NMR spectra of (a) 4AA, (b) 3h, (c) 6 h, (d) 9h, (e) 12h, (f) 15h, (g) 18h, and (h) 21h NCDs.

5. **Fig. S5** The UV-vis absorption (black line), PL emission (red line), and Photoluminescence excitation (PLE) (blue line) spectra of the NCDs in deionized water at various times, including (a) 3, (b) 6, (c) 9, (d) 12, (e) 15, (f) 18, and (g) 21 hours. A photo was taken using an iPhone X (2019).

6. **Fig. S6** PL emission spectra of NCDs at different excitation wavelengths changing from 340-400 nm for (a) 3 h, (b) 6 h, (c) 9 h, (d) 12 h, (e) 15 h, (f) 18 h, and (g) 21 h.

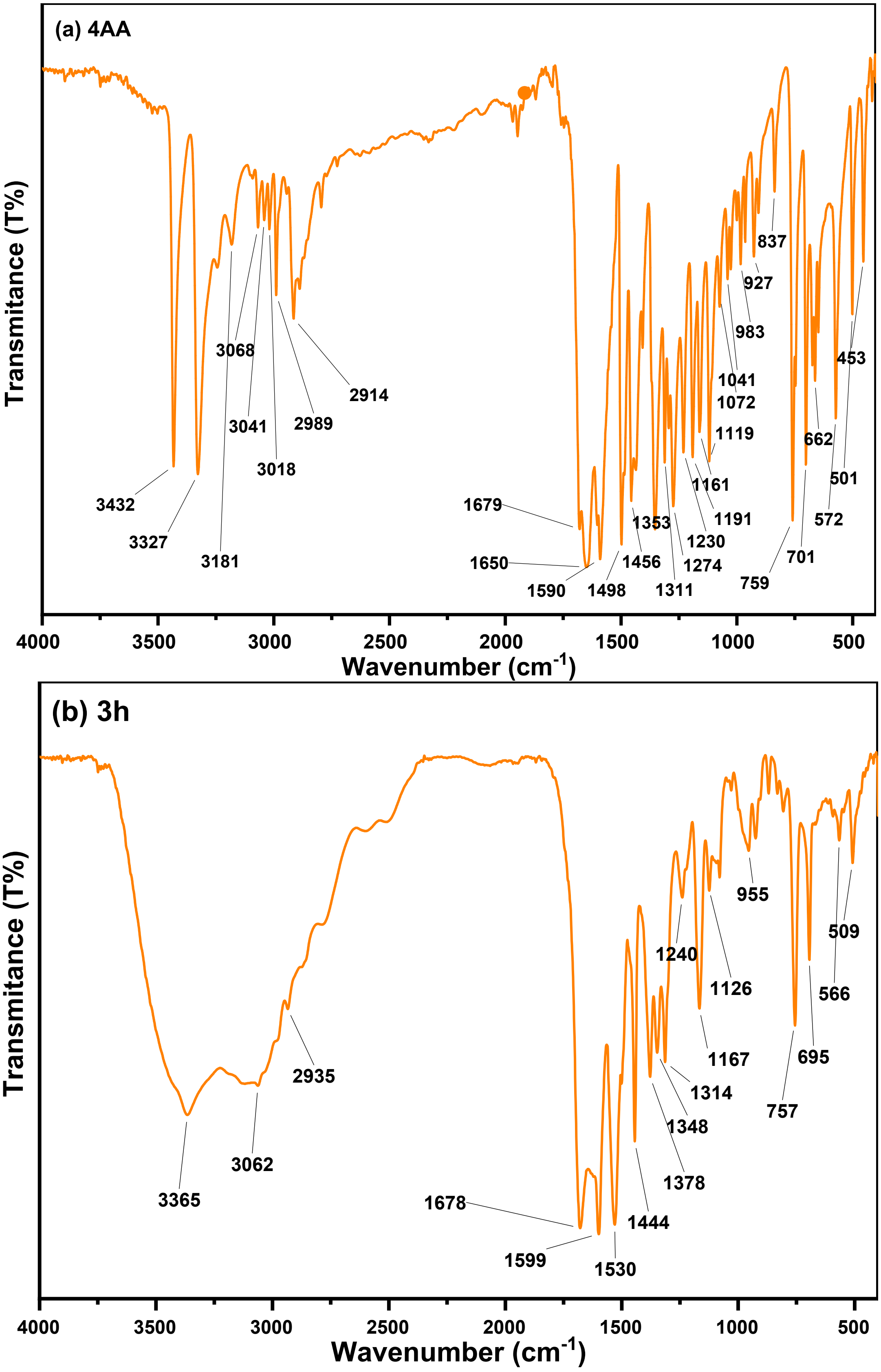
(a) 4AA
Transmitance (T%)
3432
3327
3181
3068
3041
3018
2989
2914
1679
1650
1590
1498
1456
1353
1311
1274
1230
1191
1161
1119
1072
1041
983
927
837
759
701
662
572
501
453
4000
3500
3000
2500
2000
1500
1000
500
Wavenumber (cm-1)
(b) 3h
Transmitance (T%)
3365
3062
2935
1678
1599
1530
1444
1378
1348
1314
1240
1167
1126
955
757
695
566
509
4000
3500
3000
2500
2000
1500
1000
500
Wavenumber (cm-1)

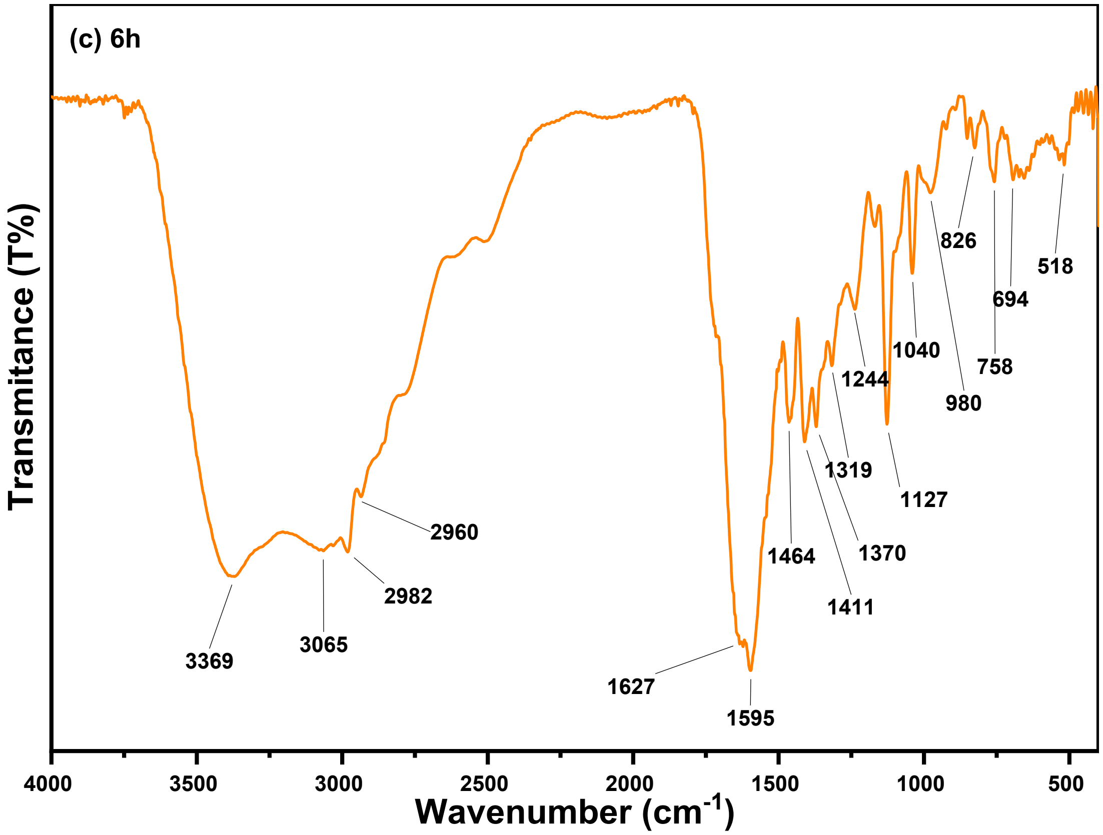

(c) 6h
Transmitance (T%)
Wavenumber (cm-1)
4000
3500
3000
2500
2000
1500
1000
500
3369
3065
2982
2960
1627
1595
1464
1411
1370
1319
1244
1127
1040
980
826
758
694
518

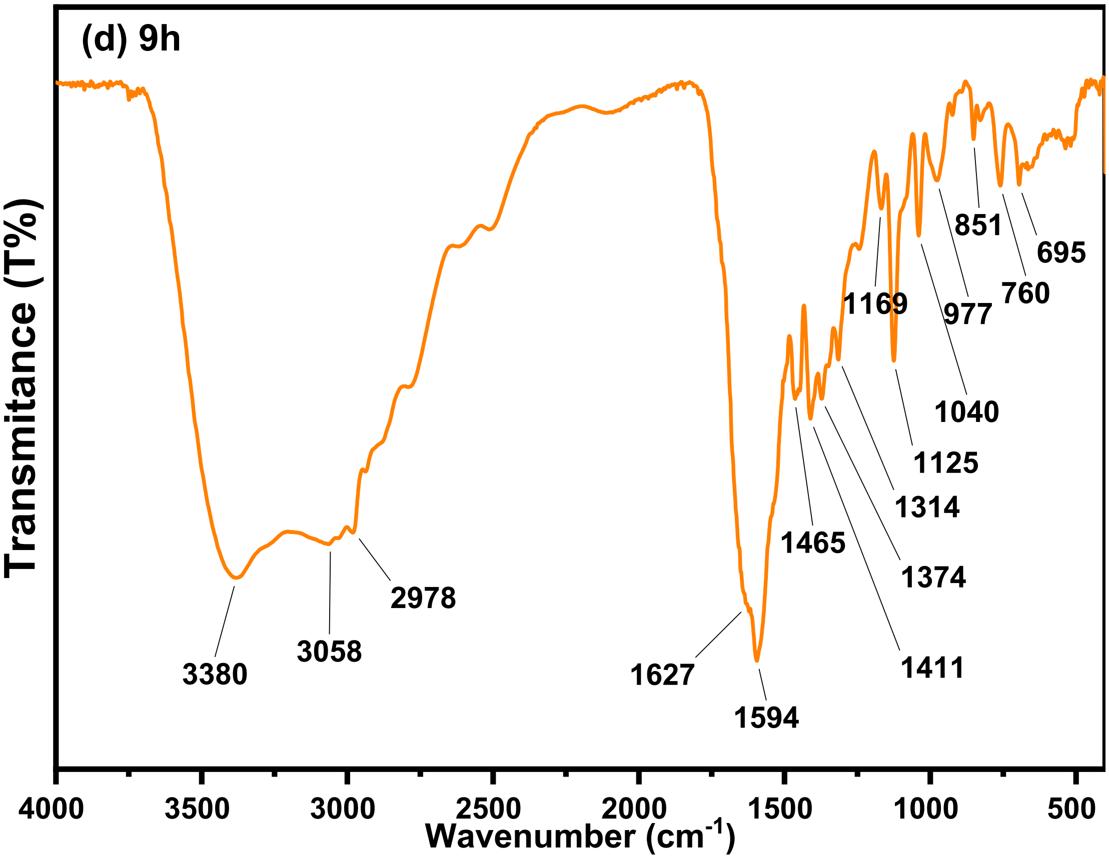

(d) 9h
Transmitance (T%)
Wavenumber (cm-1)
4000
3500
3000
2500
2000
1500
1000
500
3380
3058
2978
1627
1594
1465
1411
1374
1314
1169
1125
1040
977
851
760
695

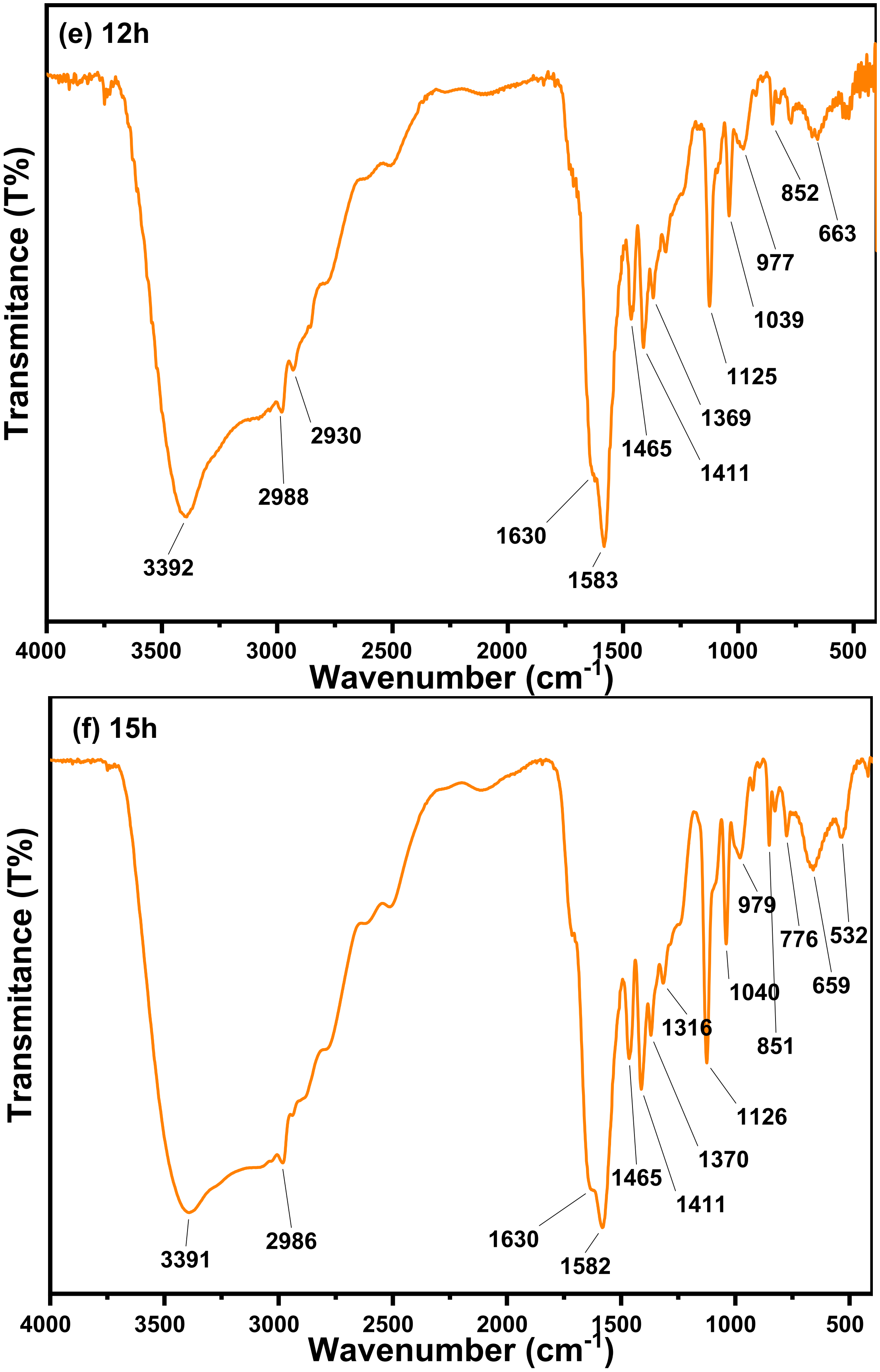
(e) 12h
Transmitance (T%)
Wavenumber (cm-1)
4000
3500
3000
2500
2000
1500
1000
500
3392
2988
2930
1630
1583
1465
1411
1369
1125
1039
977
852
663
(f) 15h
Transmitance (T%)
Wavenumber (cm-1)
3391
2986
1630
1582
1465
1411
1370
1316
1126
1040
851
979
776
659
532

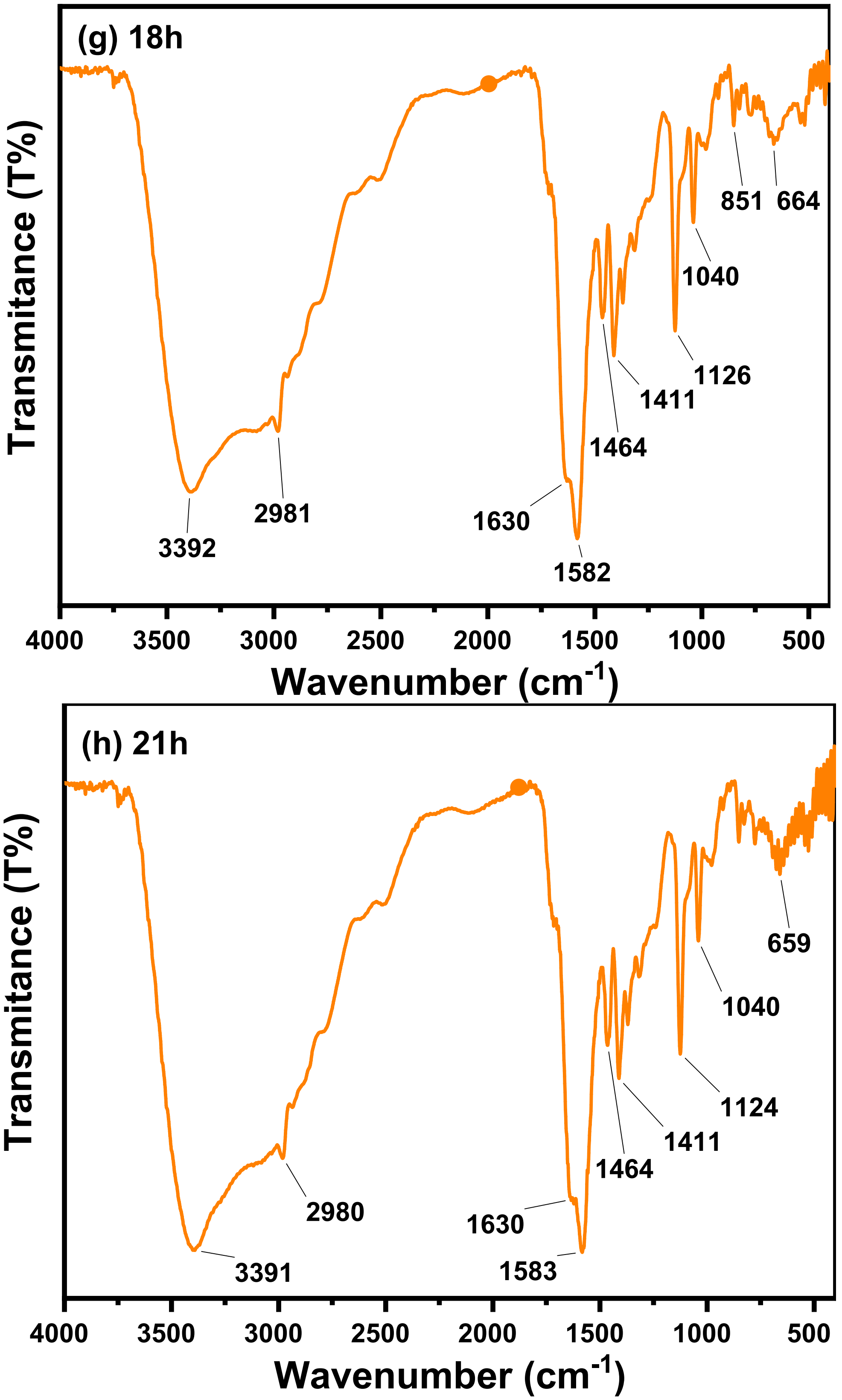

Fig. S1 FT-IR spectra of (a) 4AA, (b) 3h, (c) 6 h, (d) 9h, (e) 12h, (f) 15h, (g) 18h, and (h) 21h NCDs.

KBr压片法

FTIR spectrum of serine

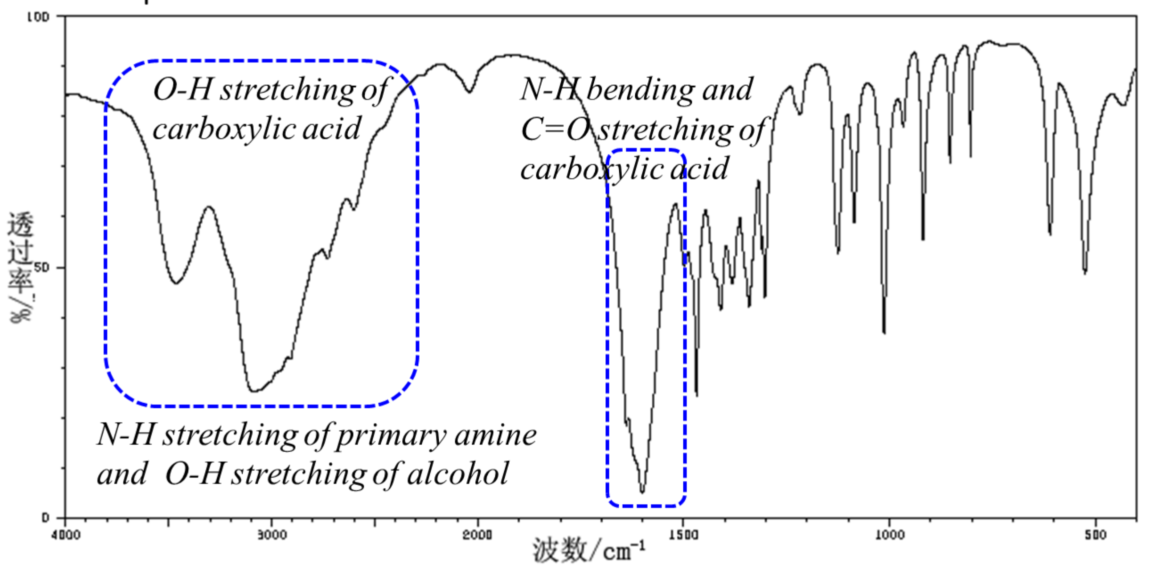

3466	44	1600	4	1303	42	863	68
3083	23	1499	49	1218	77	804	70
3072	24	1470	25	1126	50	611	59
2729	49	1411	39	1086	67	626	46
2601	58	1383	44	1014	35	434	79
2040	81	1342	41	967	74		
1640	17	1312	62	919	63		

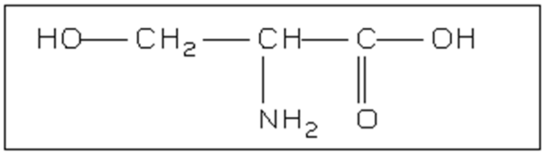

KBr压片法 FTIR spectrum of D-Threonine

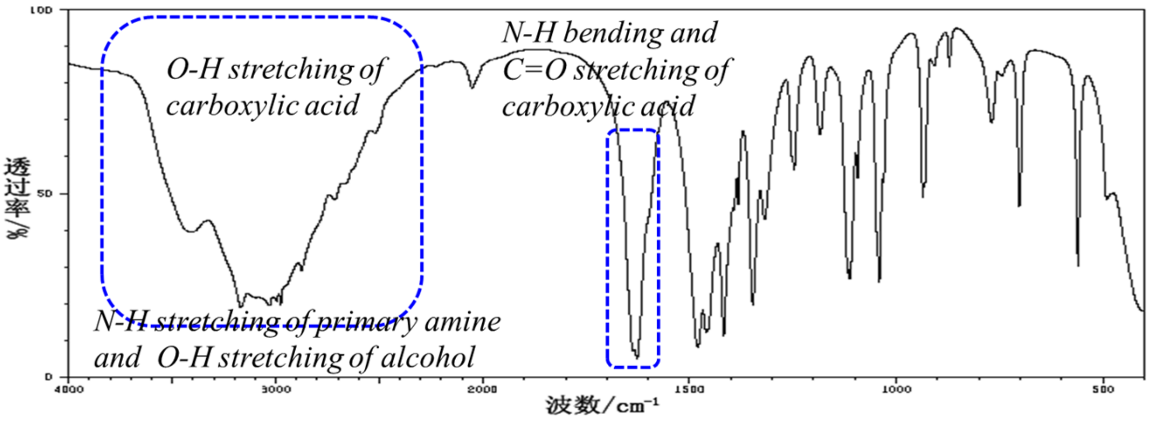

3416	37	2716	46	1382	44	1112	26	872	81
3405	37	2051	77	1348	18	1094	52	770	66
3169	16	1637	6	1319	41	1041	24	746	79
3029	18	1627	4	1263	67	1031	60	703	44
2995	20	1480	7	1247	53	937	47	561	28
2976	16	1459	11	1165	84	931	50	492	46
2874	27	1418	10	1118	26	908	81		

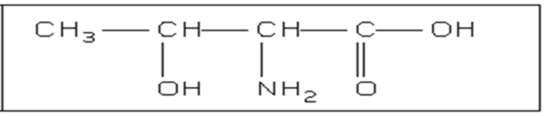

Fig. S2 FT-IR spectra of (a) serine, and (b) d-threonine.

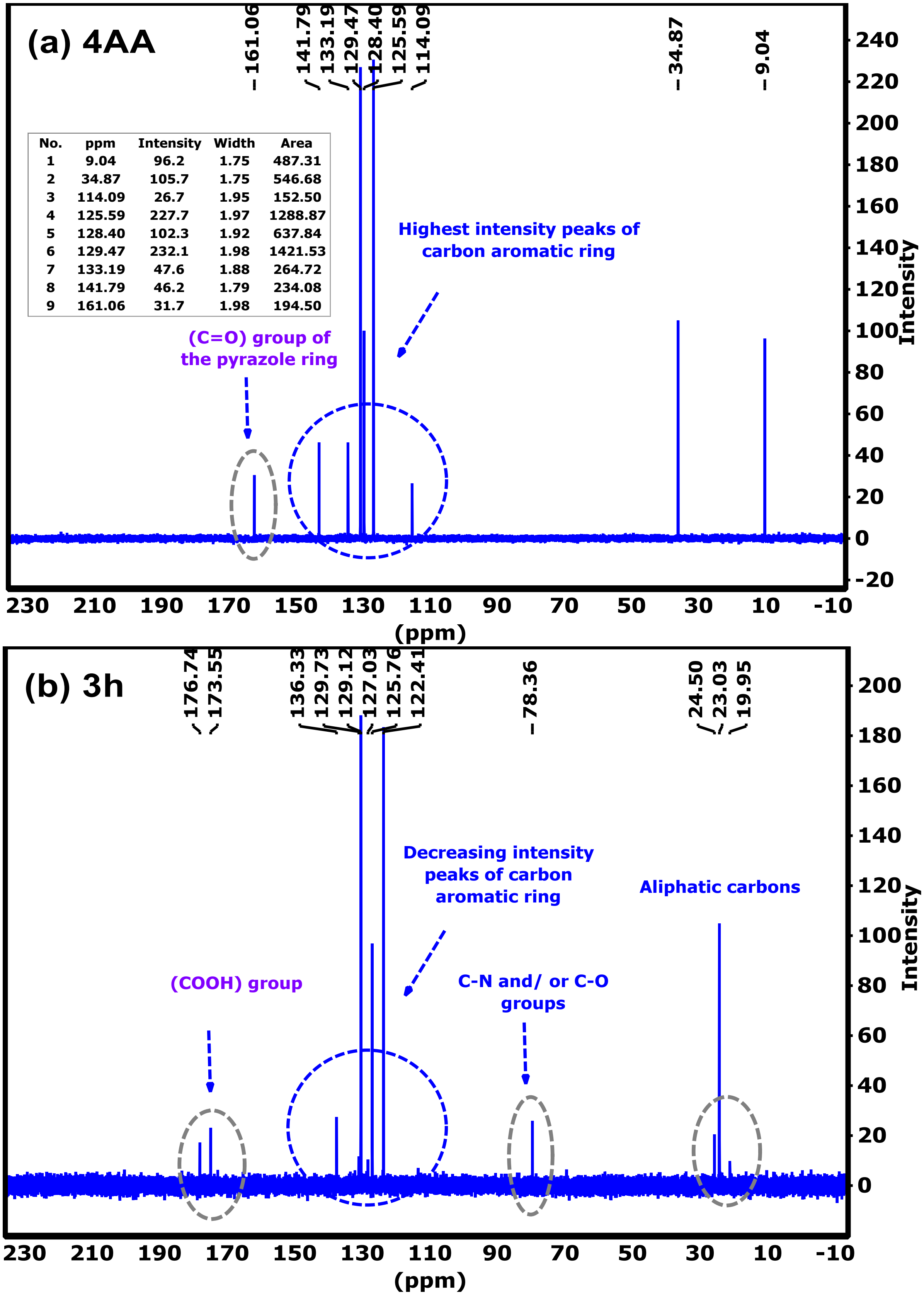

No.	ppm	Intensity	Width	Area
1	9.04	96.2	1.75	487.31
2	34.87	105.7	1.75	546.68
3	114.09	26.7	1.95	152.50
4	125.59	227.7	1.97	1288.87
5	128.40	102.3	1.92	637.84
6	129.47	232.1	1.98	1421.53
7	133.19	47.6	1.88	264.72
8	141.79	46.2	1.79	234.08
9	161.06	31.7	1.98	194.50

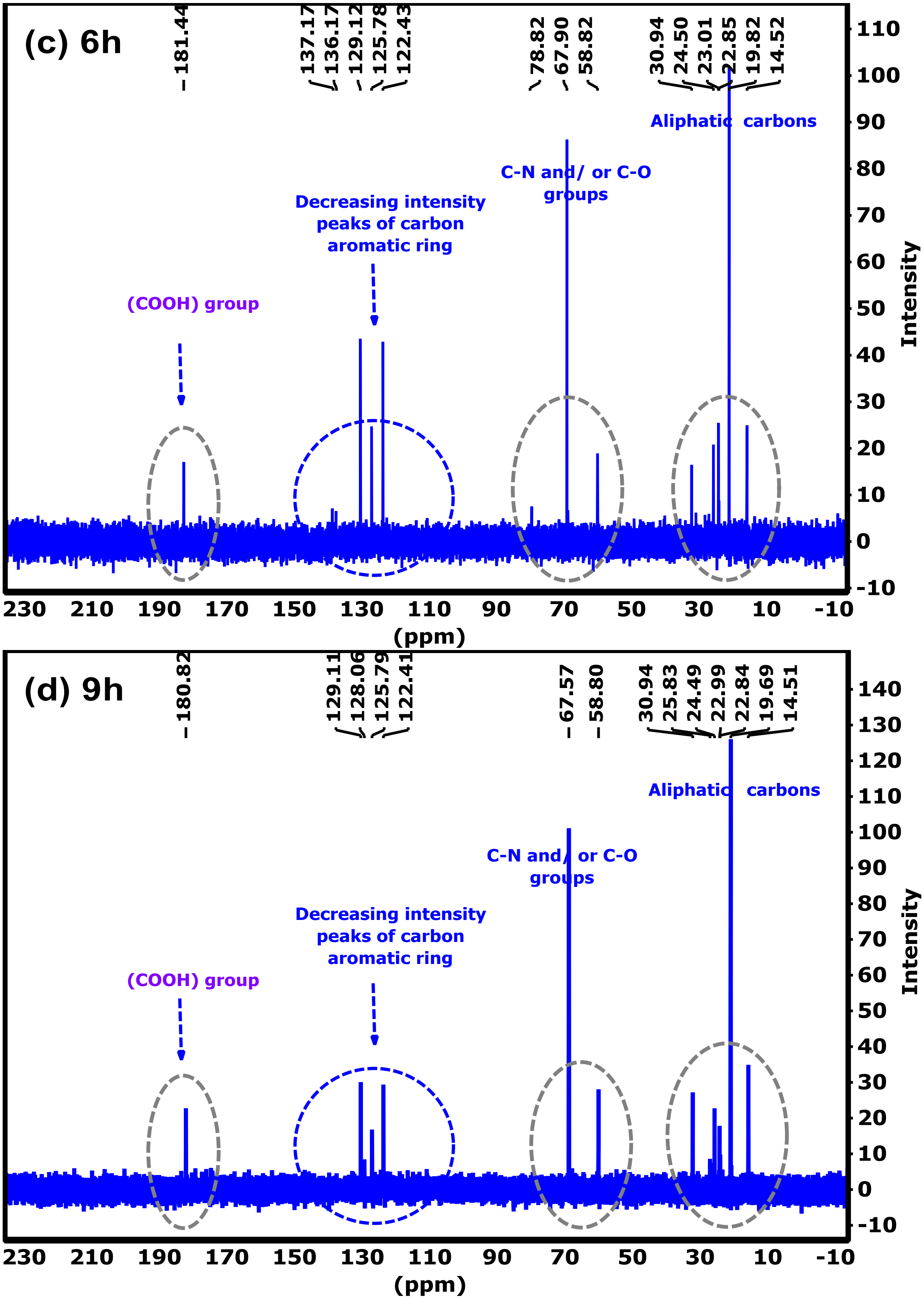

(c) 6h
181.44
137.17
136.17
129.12
125.78
122.43
78.82
67.90
58.82
30.94
24.50
23.01
22.85
19.82
14.52
Aliphatic carbons
C-N and/ or C-O groups
Decreasing intensity peaks of carbon aromatic ring
(COOH) group
Intensity
(ppm)
(d) 9h
180.82
129.11
128.06
125.79
122.41
67.57
58.80
30.94
25.83
24.49
22.99
22.84
19.69
14.51
Aliphatic carbons
C-N and/ or C-O groups
Decreasing intensity peaks of carbon aromatic ring
(COOH) group
Intensity
(ppm)

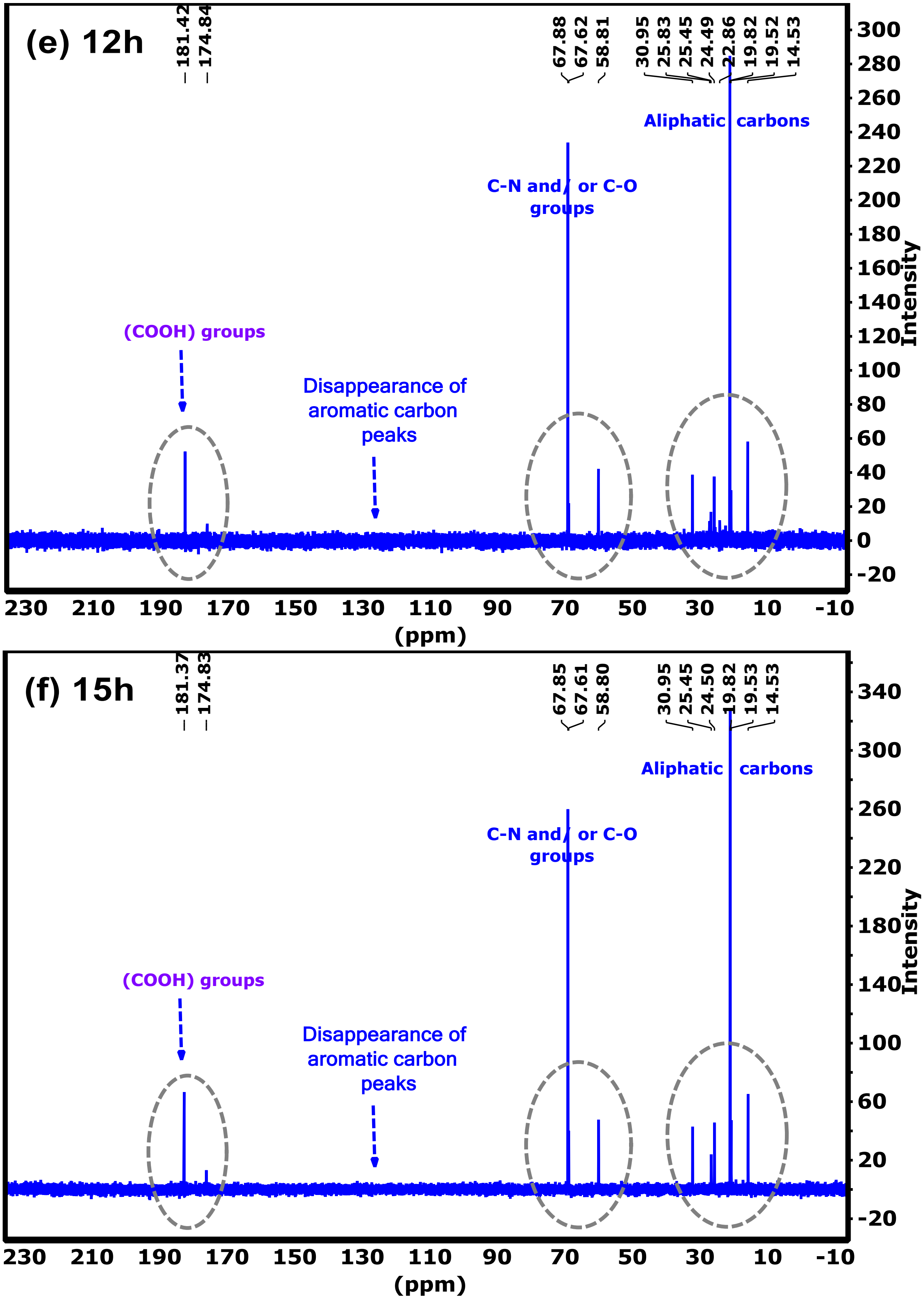

(e) 12h
181.42
174.84
67.88
67.62
58.81
30.95
25.83
25.45
24.49
22.86
19.82
19.52
14.53
Aliphatic carbons
C-N and/ or C-O groups
(COOH) groups
Disappearance of aromatic carbon peaks
Intensity
(ppm)
(f) 15h
181.37
174.83
67.85
67.61
58.80
30.95
25.45
24.50
19.82
19.53
14.53
Aliphatic carbons
C-N and/ or C-O groups
(COOH) groups
Disappearance of aromatic carbon peaks
Intensity
(ppm)

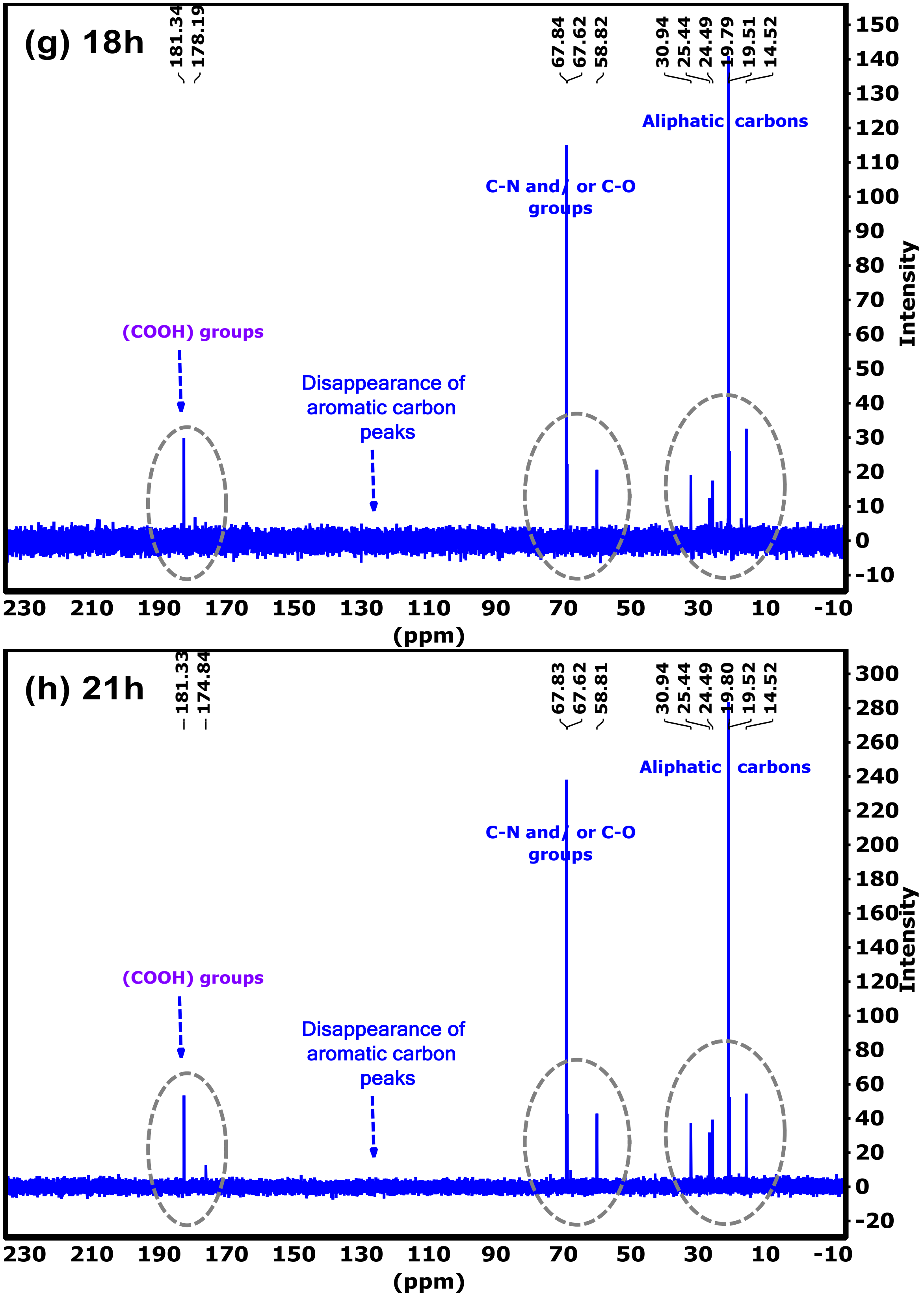

Fig. S3 ^{13}**C-NMR spectra of (a) 4AA, (b) 3h, (c) 6 h, (d) 9h, (e) 12h, (f) 15h, (g) 18h, and (h) 21h NCDs.**

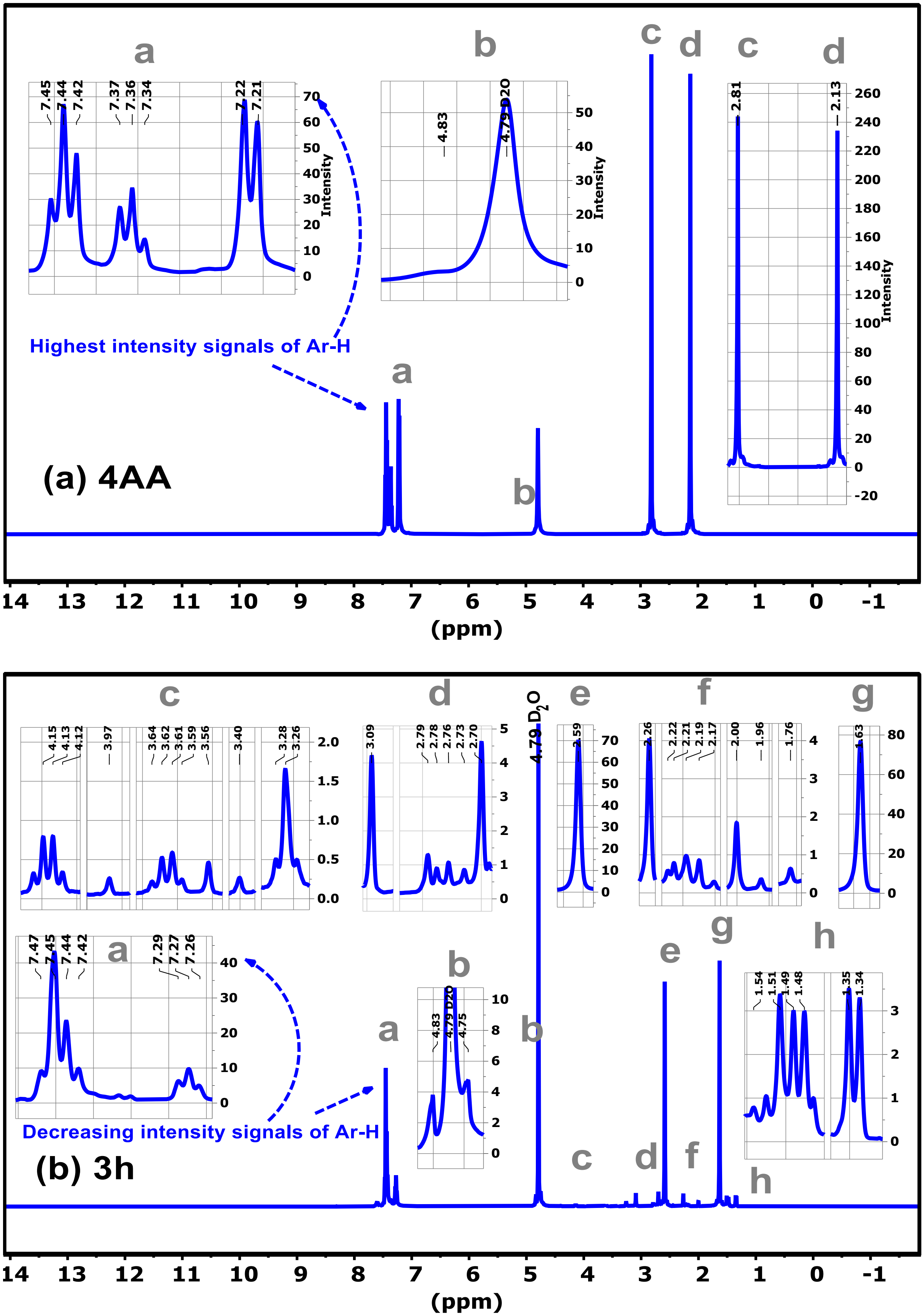

(a) 4AA
Highest intensity signals of Ar-H
a
b
c
d
7.45
7.44
7.42
7.37
7.36
7.34
7.22
7.21
4.83
4.79 D2O
2.81
2.13
Intensity
(ppm)
(b) 3h
Decreasing intensity signals of Ar-H
a
b
c
d
e
f
g
h
4.79 D2O
7.47
7.45
7.44
7.42
7.29
7.27
7.26
4.83
4.75
4.15
4.13
4.12
3.97
3.64
3.62
3.61
3.59
3.56
3.40
3.28
3.26
3.09
2.79
2.78
2.76
2.73
2.70
2.59
2.26
2.22
2.21
2.19
2.17
2.00
1.96
1.76
1.63
1.54
1.51
1.49
1.48
1.35
1.34
(ppm)

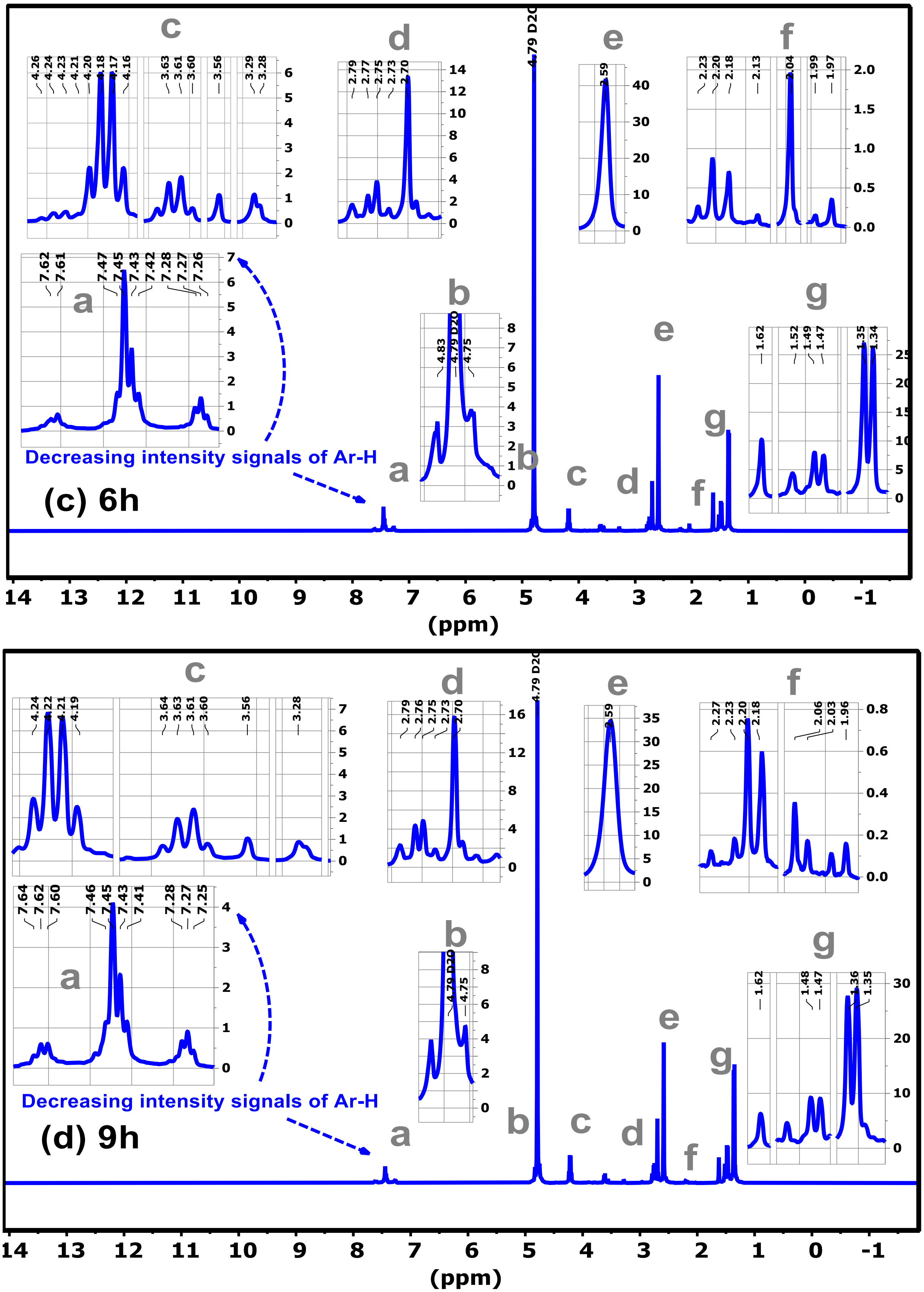

(c) 6h
Decreasing intensity signals of Ar-H
4.79 D2O
a
b
c
d
e
f
g
(ppm)
(d) 9h
Decreasing intensity signals of Ar-H
4.79 D2O
a
b
c
d
e
f
g
(ppm)

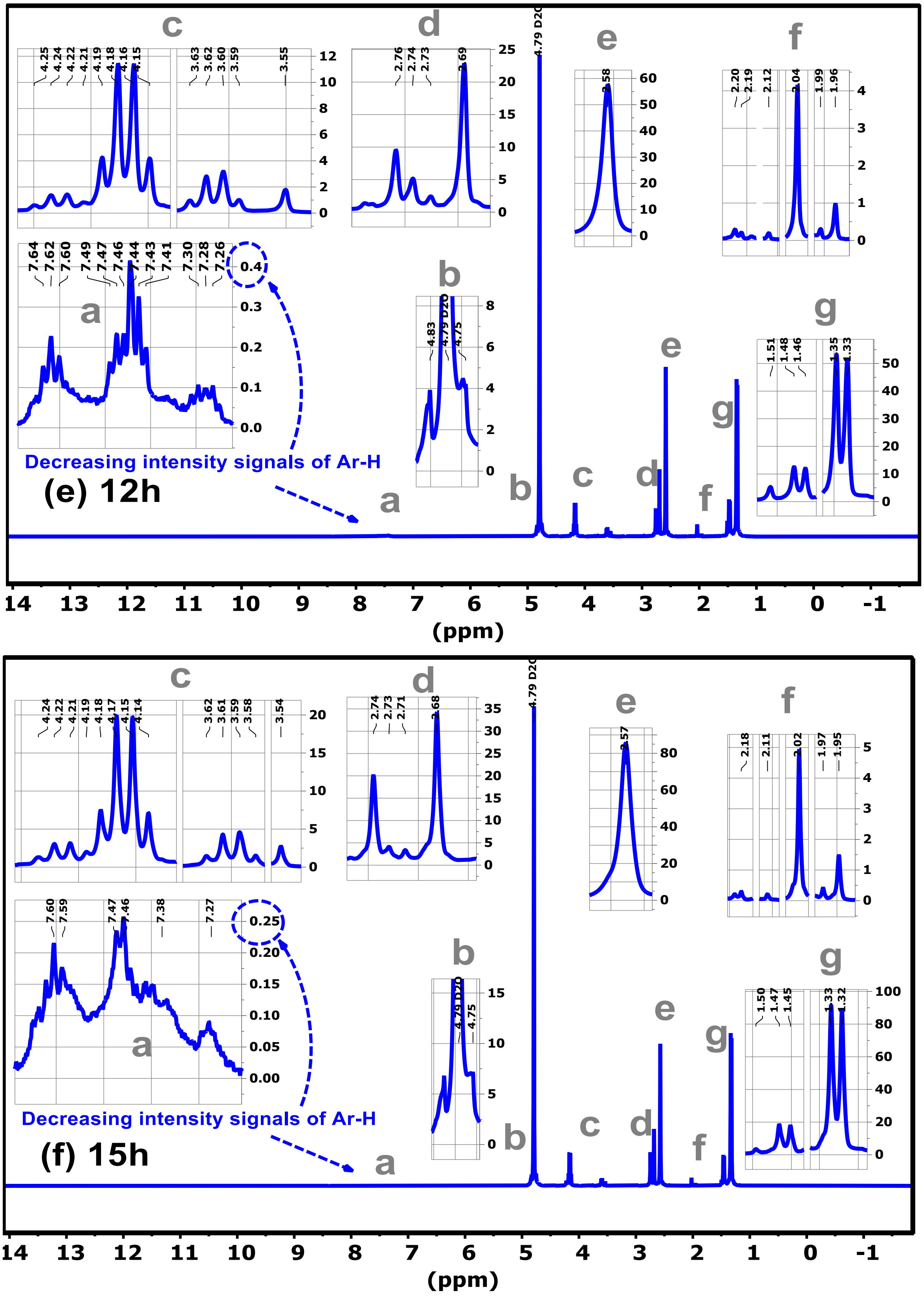
(e) 12h
Decreasing intensity signals of Ar-H
a
b
c
d
e
f
g
4.79 D2O
7.64 7.62 7.60 7.49 7.47 7.46 7.44 7.43 7.41 7.30 7.28 7.26
4.83 4.79 D2O 4.75
4.25 4.24 4.22 4.21 4.19 4.18 4.16 4.15 3.63 3.62 3.60 3.59 3.55
2.76 2.74 2.73 2.69
2.58
2.20 2.19 2.12 2.04 1.99 1.96
1.51 1.48 1.46 1.35 1.33
(ppm)
(f) 15h
Decreasing intensity signals of Ar-H
a
b
c
d
e
f
g
4.79 D2O
7.60 7.59 7.47 7.46 7.38 7.27
4.79 D2O 4.75
4.24 4.22 4.21 4.19 4.18 4.17 4.15 4.14 3.62 3.61 3.59 3.58 3.54
2.74 2.73 2.71 2.68
2.57
2.18 2.11 2.02 1.97 1.95
1.50 1.47 1.45 1.33 1.32
(ppm)

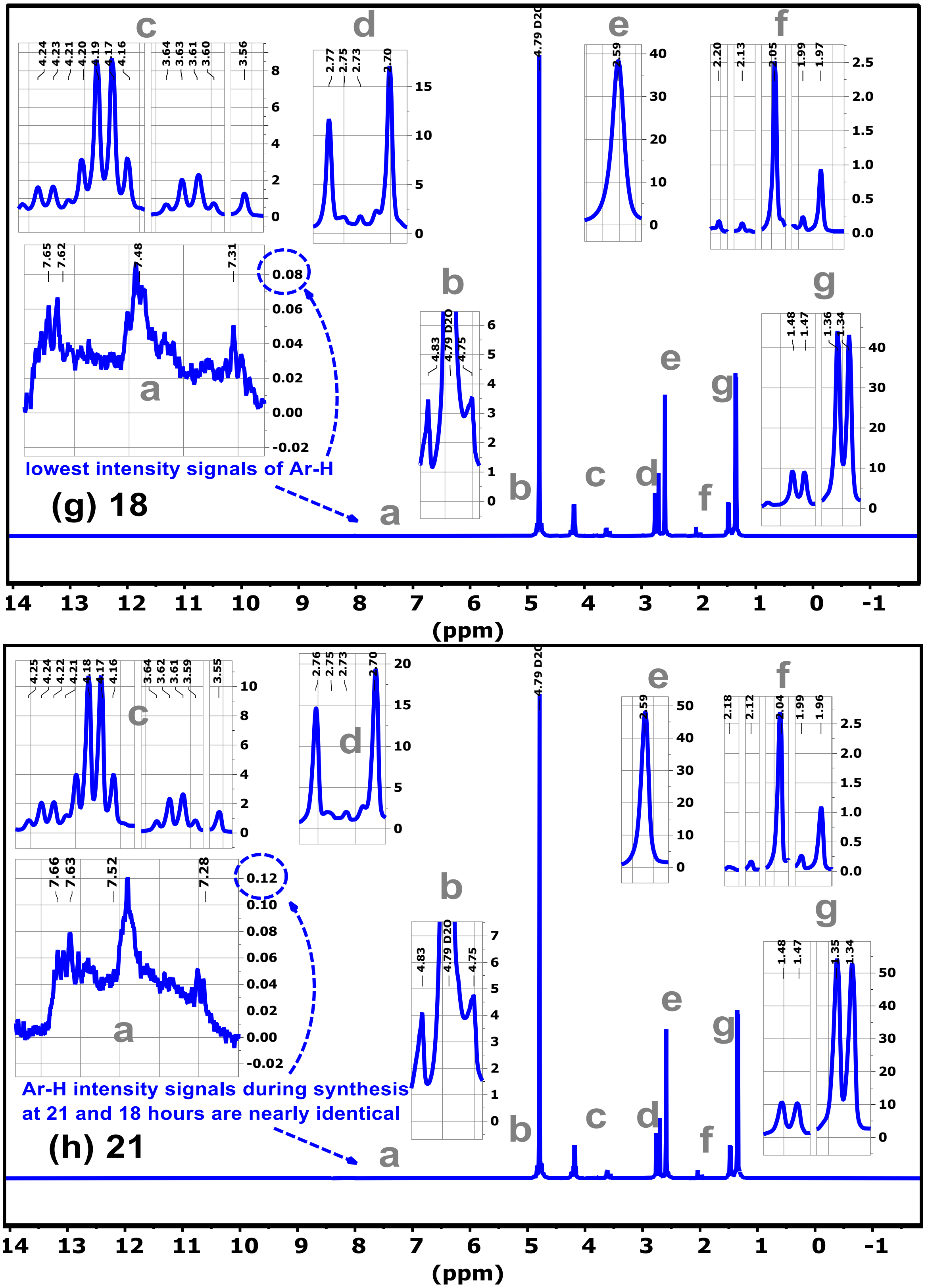

Fig. S4 ^{1}H-NMR spectra of (a) 4AA, (b) 3h, (c) 6 h, (d) 9h, (e) 12h, (f) 15h, (g) 18h, and (h) 21h NCDs.

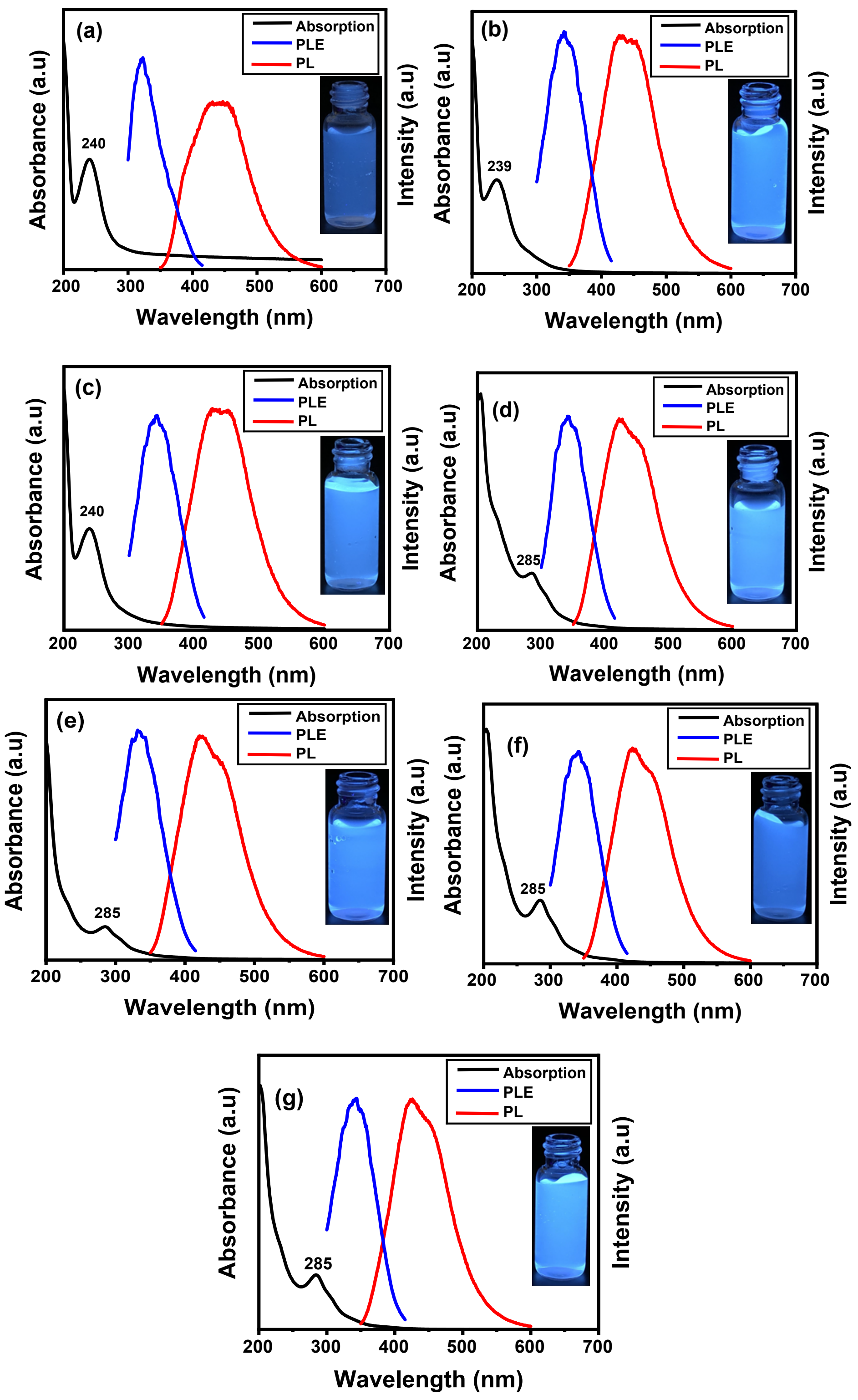

Fig. S5 The UV-vis absorption (black line), PL emission (red line), and Photoluminescence excitation (PLE) (blue line) spectra of the NCDs in deionized water at various times, including (a) 3, (b) 6, (c) 9, (d) 12, (e) 15, (f) 18, and (g) 21 hours. A photo was taken using an iPhone X (2019).

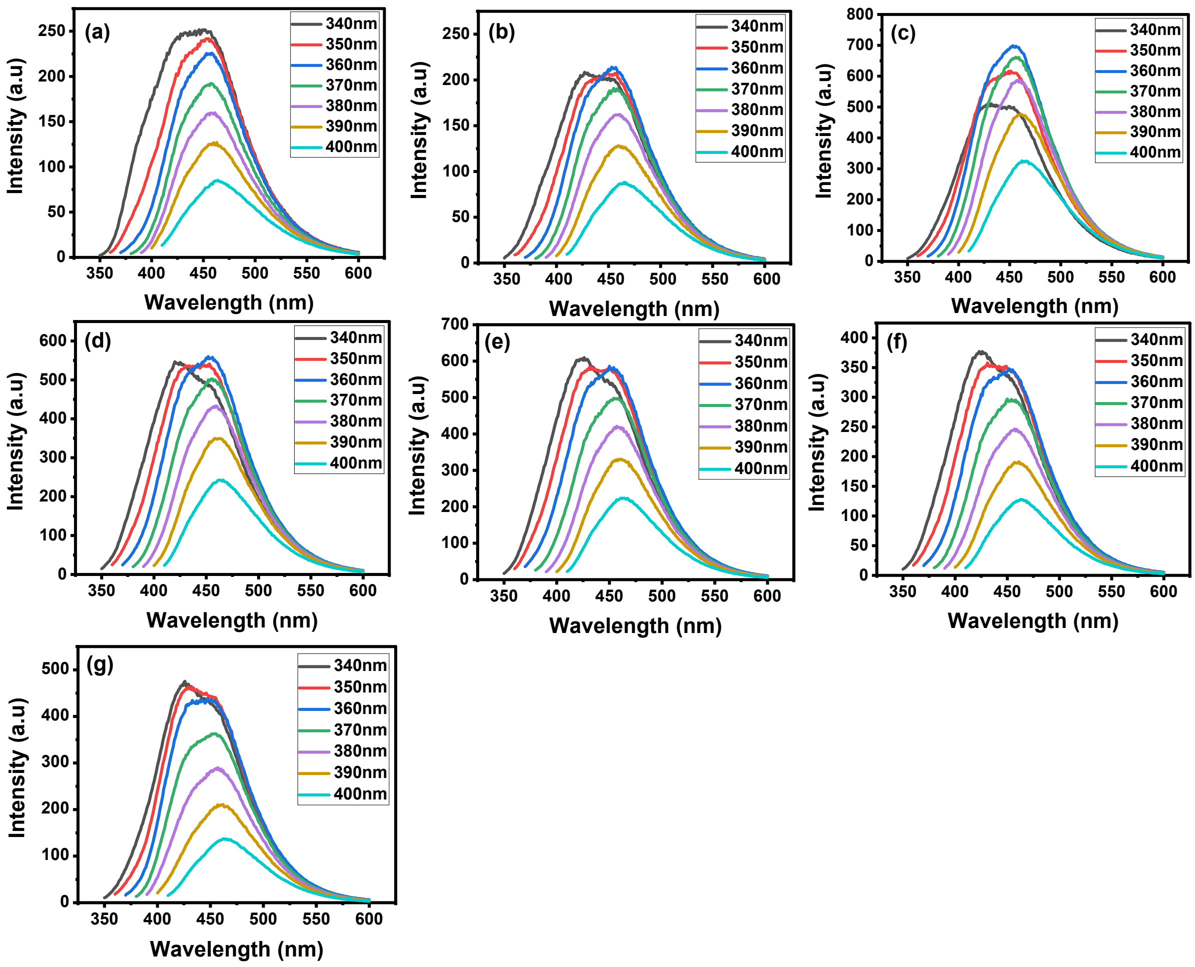

Fig. S6 PL emission spectra of NCDs at different excitation wavelengths changing from 340-400 nm for (a) 3 h, (b) 6 h, (c) 9 h, (d) 12 h, (e) 15 h, (f) 18 h, and (g) 21 h.
